# Lawson Wilkins: recollections by his daughter

**DOI:** 10.1186/1687-9856-2014-S1-S1

**Published:** 2014-05-28

**Authors:** Elizabeth Wilkins McMaster

**Affiliations:** 1 135 Lloyd Avenue, Providence, Rhode Island 02906, USA

## Abstract

Lawson Wilkins is well known as the “father” of the field of pediatric endocrinology, and his scientific accomplishments and legacy are thoroughly documented in this edition and elsewhere. Less well known, though, is what the man himself was like. Here, his daughter, Elizabeth McMaster, recalls the personal side of Dr. Wilkins including his upbringing as the son of a prominent Baltimore doctor, his medical education, establishment of a successful pediatric practice, and eventually the founding of the endocrine clinic at Johns Hopkins.

Interwoven with anecdotes and reminiscences, this account provides a vivid sense of Wilkins’ personality and life, from his boisterous nature and devotion to his family and career, to the tragic personal losses he endured. He was a man who threw himself fully into everything he did, whether it was making his own liqueur during Prohibition, collecting specimens from abnormally large circus performers as part of his earliest endocrine research, arranging raucous, impromptu singing parties, sailing the Chesapeake with friends, writing a definitive textbook of Pediatric Endocrinology, training a legion of fellows, or the pioneering work for which he is still known today.

## Introduction and early life

From my earliest childhood I always knew that my father, Lawson Wilkins, was a truly remarkable person. Unlike my friends’ fathers who went to offices and did some vague kind of work, my father worked long and irregular hours at the most important work in the world—taking care of sick children, saving lives and doing detective work into mysterious illnesses. He relished hard work and mastering new subjects of all sorts, wanting to know all there was to know about the history or archeology of a place he was to visit, opera, whatever his children were studying. In his rare spare time, instead of playing golf, he liked to plunge into grueling work in his garden, ambitious cabinet work or ship model making. His favorite vacations before World War II were spent with friends, sailing the wilder regions of Chesapeake Bay, having exciting and fabulous adventures that became fodder for family legends and songs. He had a great range of friends and relished parties at home where, notoriously untalented in music, he would sing or play the violin with gusto or engage in hilarious, elaborately staged charades. He was a very loving father who made the brief vacations and days off memorable with raucous card games, patient lessons in fishing, sailing and swimming, and occasional excursions. Whatever he did or taught us, he demonstrated, rather than demanded, intellectual curiosity, thoroughness and one’s very best effort. Add to these qualities a hearty—at times riotous—sense of humor and an irrepressible joie de vivre—and things were not dull when he was around. Most of all, he adored his “Lu”, my mother who complemented all these qualities and truly made it possible for him to live his life as he did. And it was she who early explained to me that he was blessed to have as his core a calling that he so loved and that made it possible for him to live such a fulfilling life.

Lawson Wilkins was born on March 6, 1894, at 223 S. Broadway, Baltimore, MD. Presumably, his father, Dr. George Lawson Wilkins, an established physician, delivered him. His safe arrival must have been a cause of great rejoicing as the parents had previously lost two babies in infancy. Had they known what lay ahead for this baby, their joy and pride would have been immensely greater.

Lawson’s father, known as “George” and “Lawson,” had been born in or around Portsmouth, Virginia, in the tumultuous year of 1848. His family was said to have come from Ballyhack, a charming, small village near Waterford, on the southeast coast of Ireland. I do not know when or why they came to America. One family name was Lynch. One ancestor was reputedly named Pythagoras, perhaps a family legend. His father, Richard, was said to be an engineer; his mother’s name is uncertain—thought to be Lydia. A charming daguerreotype shows George as a small boy with his sister, Mary Alice. His serious round face resembles that of his son, born almost forty years later. Family legend was that the family lived at Hampton Roads and George could have watched the battle of the Monitor and the Merrimac there in 1862. In truth, we know little about his family and childhood. Mary Alice married Norman Etheridge and I have names of her children and grandchildren with whom Lawson apparently had no contact or lost touch. We know nothing of how the family fared during or after the Civil War.

A sheepskin diploma in Latin declares that G. Lawson Wilkins received the degree of Doctor of Medicine at the University of Maryland in Baltimore, on March 10, 1870. Although founded in 1807, as part of a land grant university, this medical school was not in the forefront of medicine at the time. In the mid-nineteenth century, with the rapid expansion westward, the demand for doctors of all sorts had outpaced the supply. Many proprietary schools, with low or non-existent standards, private businesses run by doctors, had sprung up. During the Civil War, both the city and the university were bitterly divided. This, combined with the competition, caused the medical school’s enrolment to drop. When George Wilkins attended the school, the course in medicine lasted for two or three full years, unlike some of the “diploma mills” where the course was for six weeks only. It included anatomy learned using cadavers, unlike many other schools, and the student had to write and defend a dissertation. After graduation, the new doctor did an apprenticeship of six to twelve months before setting up practice [1].

George Wilkins practiced general medicine in East Baltimore, which teemed with a variety of ethnic groups. Germans displaced by the revolutions of 1848 made up a large segment of the population, followed later by Poles, Lithuanians, Bohemians, Czechs, Italians and immigrants of many other countries, drawn to the second largest port in the United States, with its many industries. His office and home were presumably in the building at 223 South Broadway which still stands, with an unusual wrought iron porch, reminiscent of Charleston.

In addition to his private practice, Dr. Wilkins was appointed the physician at the Baltimore City Jail, a position he held for many years. Newspaper clippings from the early 1880’s contain colorful testimony he gave in proceedings to have a man accused of bigamy declared insane. An account of the death of a renowned robber in jail described the robber’s last hours conversing with Dr. Wilkins who had become quite fond of him and remained with him until his death [2].

**Figure 1:**
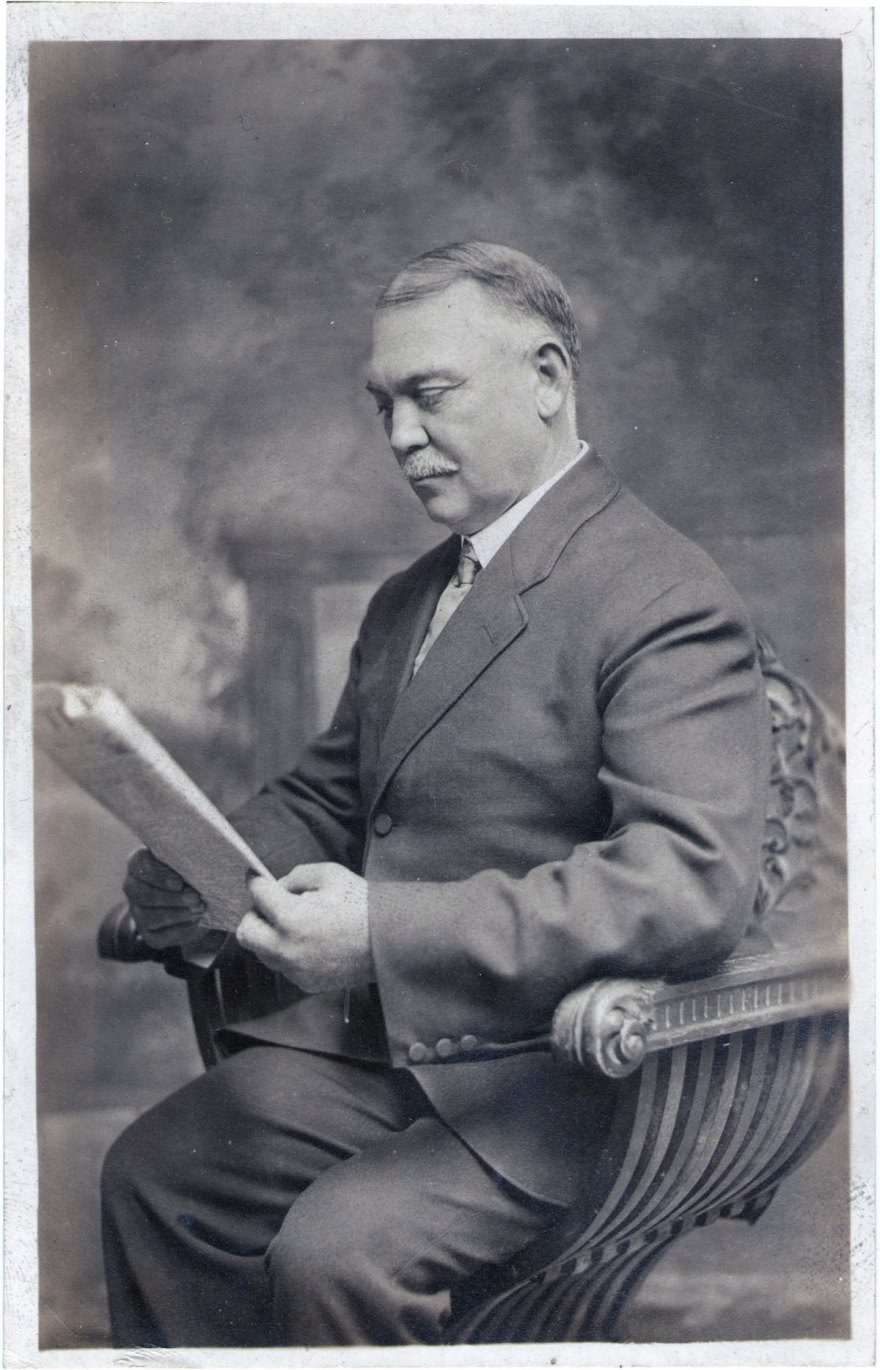
Dr. George Lawson Wilkins

By 1882, Dr. Wilkins was a very active member of the Grand Lodge of the Ancient Order of United Workmen, a secret fraternal organization whose purpose was “to embrace and give equal protection to all classes and kinds of labor, mental and physical; to strive earnestly to improve the moral, intellectual and social conditions of its members… to create a fund for the benefit of its members during sickness or other disability, and in case of death to pay a stipulated sum to such person or persons… thus enabling him to guarantee his family against want” (Figure 1).

On June 7, 1888, Dr. Wilkins married Harriet Isabel Schreiner in her mother’s home in Philadelphia. We do not know how they met but we have many more facts about her early life than we have of his. Her family, of English, French and German descent, had been in Philadelphia since at least the early nineteenth century. Her mother, Mary Louise Fougeray, born in 1828, married Henry Schreiner in 1846. Mr. Schreiner had been married before and had at least four children. He had six more with his second wife and then apparently abandoned the family. There does not seem to have been much contact between Harriet and her older siblings in later life but she remained very close to her two younger sisters, Emilie and Clara.

Hattie, as she was always called, was born October 6, 1857. She graduated from the Girls’ Normal School in Philadelphia and passed an examination qualifying to be a school principal. Life was apparently very hard for her mother and two young sisters. Hattie reportedly supported them by teaching. Emilie, five years younger, went to live in Philadelphia with an aunt and uncle, Jennie and Thomas Stokes, who were childless. Hattie was said to be quite musical and may have given piano lessons.

A photograph of the time of her marriage shows her to be a sweet-looking young woman with even features, wavy blond hair and a slight smile. She must have been tiny: when I was twelve years old, and quite thin, I was allowed to dress up in her clothes—a white satin wedding dress and black velvet riding habit. Her shoes, two inches wide, and gloves were much too small for me and by the time I was thirteen I had outgrown the lovely dresses.

George and Hattie lived at 223 South Broadway. There is no indication from letters of any contact with either of their families or visits from them except for Hattie’s two younger sisters, and a few letters from cousins. There are many letters from Hattie’s dear friend, Annie Baird Beaton, eleven years her junior, with whom she had been an active member of Bethany Presbyterian Church in Philadelphia. John Wanamaker, founder and owner of Wanamaker’s department store, was superintendent of the Sunday school and took a lifelong interest in these two hardworking young women. George immediately became a dear friend of Annie’s, too. When Hattie wrote to Annie, there was often a long postscript, signed “Doctor”, “Lawson” or “Fadie” from him. These reveal a droll and affectionate sense of humor.

My grandfather always spoke adoringly of Hattie and they were apparently very happy together. Letters describe Hattie teaching a Sunday school class of boys, as she had in Philadelphia, chairing the Mission Board at Second Presbyterian Church and serving on the Board of Managers of the Presbyterian Home in 1889-90. She and the doctor belonged to a social club of professional people that met biweekly for socialization and education, such as lectures and music. Occasionally friends and relatives came from Philadelphia for visits but there is only one mention of a visit from Hattie’s mother and Hattie sometimes cautioned a correspondent not to let her mother know she had been to Philadelphia without visiting her and that her mother did not like some of her friends.

Their first child, Louise Person, named for a family friend, died in August, 1891, reportedly of dysentery. Their second baby, Russell Miller, named for a pastor of Hattie’s church in Philadelphia, was born June 17, 1892. A letter to Annie, dated February 6, 1893, from George reports that Russell had bronchopneumonia, that he feared it was “the forerunner of whooping cough” and not to tell Hattie’s mother, so as not to worry her. A few days later the baby died and there are many letters of grief between the two friends.

At this time, when Baltimore and Philadelphia, among other cities, had open sewers and unsafe water supplies, when pediatrics did not exist as a specialty and medicine had little to offer for childhood diseases, infant mortality was very high. For Hattie, now aged thirty-six and George, forty-five, the grief must have been profound. There are many letters from sympathetic relatives and friends, notably Hattie’s friend and mentor, John Wanamaker, and the beloved minister, Russell Miller. Many expressed the view, voiced by Mr. Miller that “A child in heaven is a more sacred tie than a child on earth” and that Hattie should think of Russell’s death as “another of God’s beautiful lessons. He is preparing you for a new and larger and sweeter service in the future. He has such training as this only when he is fitting his disciples for most delicate and honorable work. Your grief and sorrow have prepared your heart and hand for ministering to others in the days to come.” Perhaps these were comforting words to Hattie, who obviously was a very devout woman, but that is not the view her son Lawson would have when he grew up; he could not believe a compassionate God inflicted such pain for such a purpose. In any case, Hattie had a very loving, sensitive and compassionate husband and they were soon to know greater happiness.

**Figure 2:**
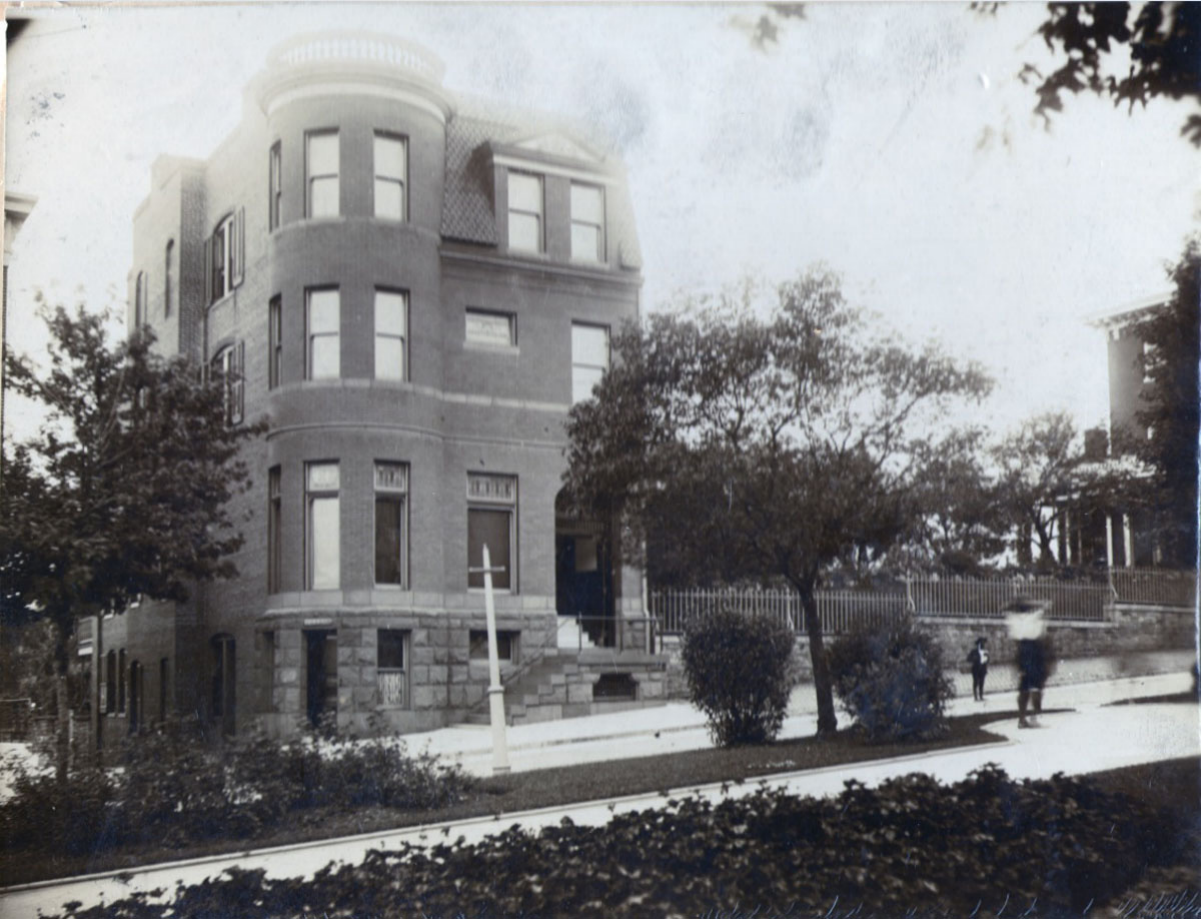
6 North Broadway, home of Dr. George Lawson Wilkins

In 1893, my grandfather built an imposing four-story brick house almost on the crest of the hill at 6 North Broadway, just south of Johns Hopkins Hospital, with a stone foundation, corner turret with a balustrade on top, separate entrance to his medical office and a large garden behind it that made a statement of success and comfort. Photographs of the period show ornate dark woodwork, gas-lit brass chandeliers, flocked wallpaper and formal Victorian furniture. At that time, Broadway was a fine place to live, (in spite of the sewers) with fountains in the grassy median and Patterson Park nearby. When I passed by in March, 2013, the house appeared to have been rehabilitated and was for sale. Since the 1940’s, it had been a boarding house, a rooming house and then vacant for years (Figure 2).

**Figure 3:**
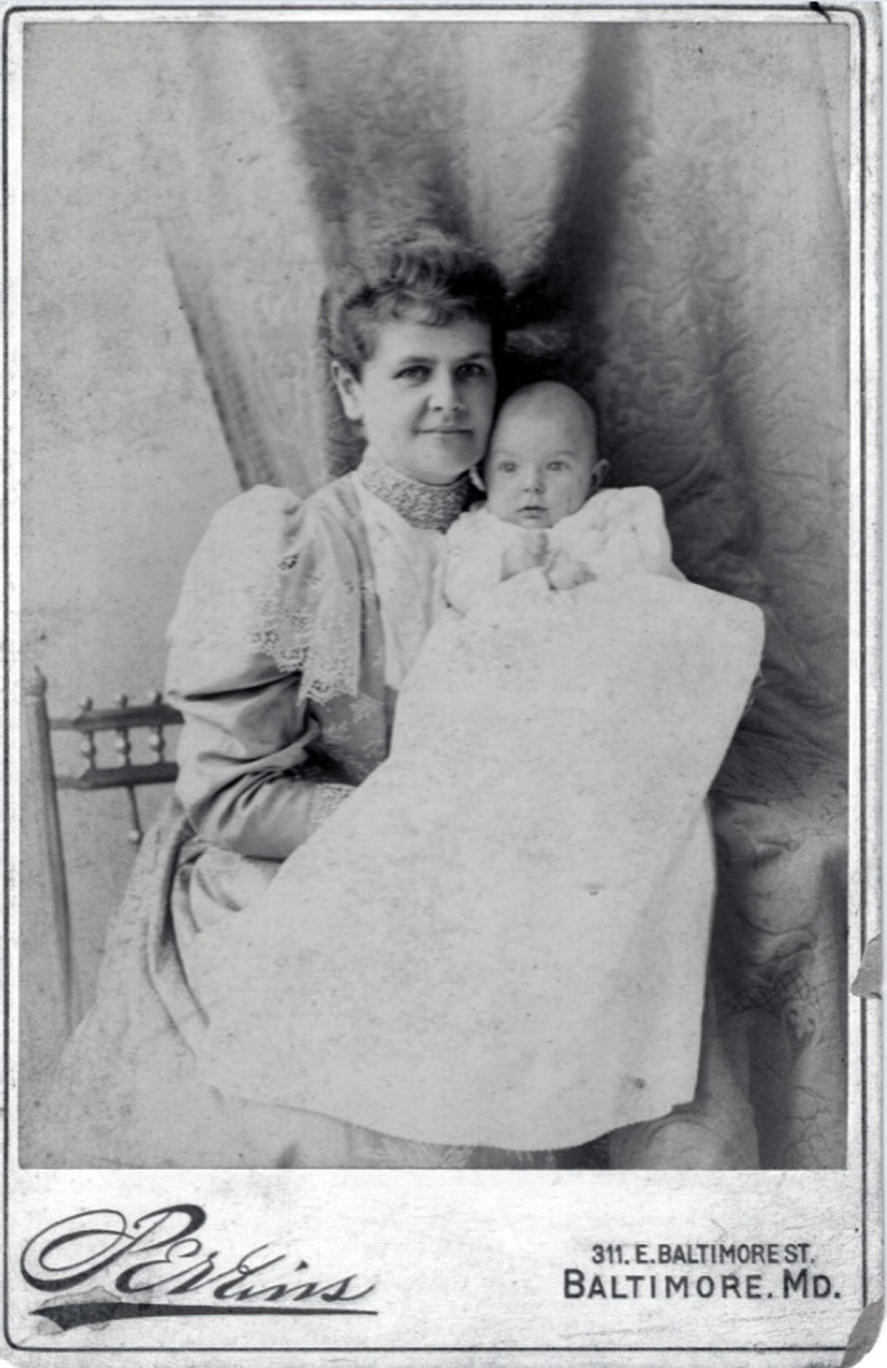
Harriet S. Wilkins with baby Lawson

**Figure 4:**
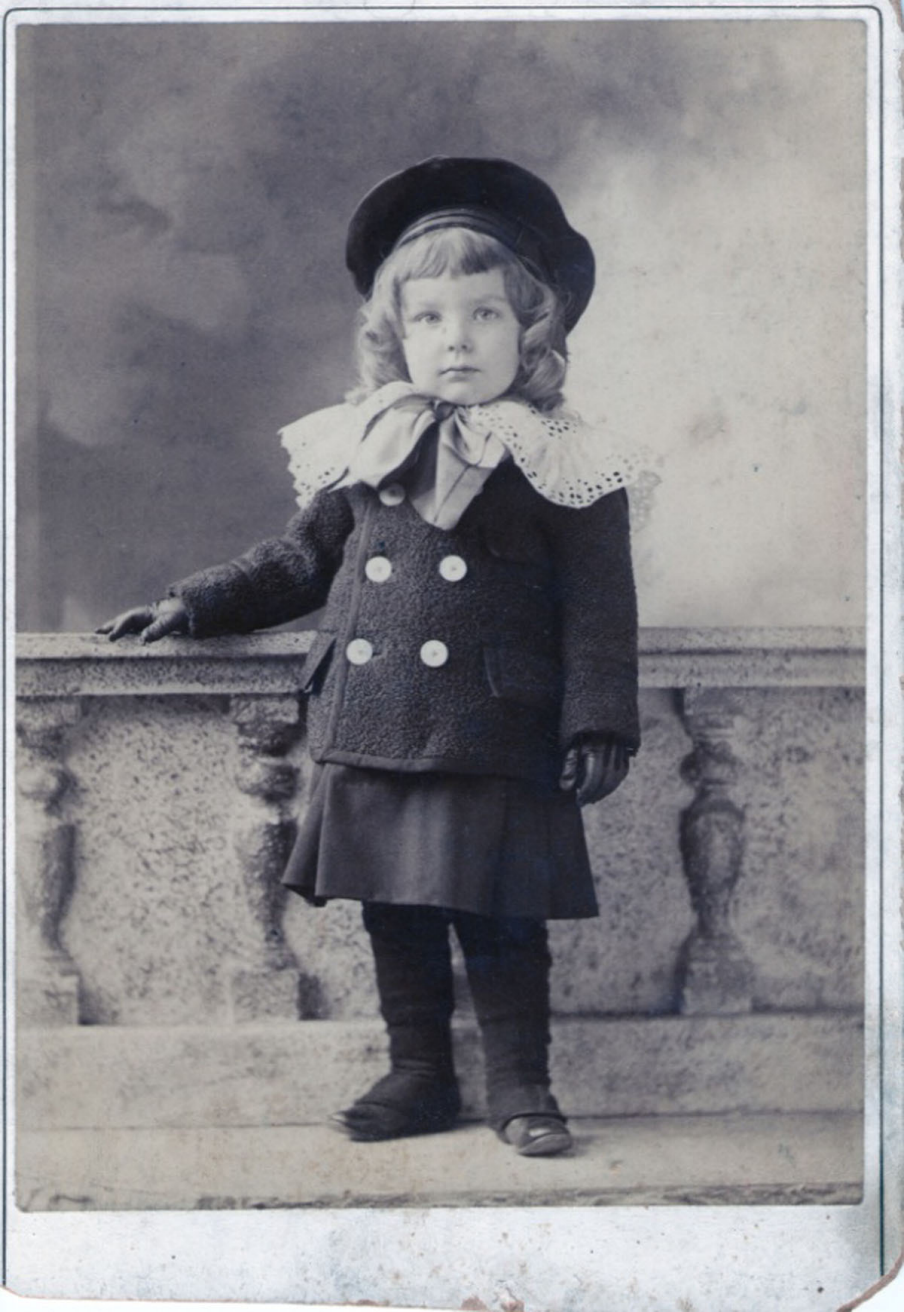
Lawson Wilkins age 3

Lawson’s birth on March 6, 1894, and healthy infancy must have seemed a God-sent joy to these loving parents. I know very little of his early childhood, except that he had happy memories of his parents. There were several servants and usually some hunting dogs as pets. In 1897, his sister, Emilie, was born. One of his early memories he humorously described as “having broken a leg in the Spanish-American War.” As he was running down a long, highly polished hall, he collided with the incumbent pet, Lena a Pointer—and fell (Figures 3 &4).

In 1900, Hattie was pregnant again. My grandfather later reported how thrilled Hattie was to have another baby but soon this happy family was plunged in grief. Six or seven months pregnant, Hattie was operated on for appendicitis. Shortly thereafter she was operated on again and the baby was taken. While apparently recovering, she developed septicemia and died. The renowned Drs. Halsted and Kelly had attended her. Lawson always grieved for the dear mother he had known. Emilie described a lifelong yearning for the mother she could not remember. No one has reported how my grandfather described his feelings at losing this patient, his wife, whom, like their first two babies, his medical skill could not save.

## A childhood of sadness and success

During Hattie’s confinements, losses of the babies and infancies of Lawson and Emilie, Hattie’s sister, Clara, and their dear friend, Annie Beaton, spent time with her in Baltimore, although Clara worked for some years as a missionary teacher with Indians in Tucson, Arizona, and Annie had an office job with a land title company in Philadelphia.

Into this somber atmosphere, Aunt Clara Schreiner came to care for the bereaved children. Aside from her missionary work out west, there is no record of what work she had done. She had several mental breakdowns, apparently before and definitely after this period. According to my father, she subscribed to a very Calvinistic, “Hellfire and brimstone” form of Presbyterianism. There was little joy in the home.

Two factors redeemed Lawson’s childhood from absolute gloom. His father, whom those who knew him described as very similar to his son in adult life, was a wonderful companion to the little boy, teaching him carpentry and taking him hunting and fishing in nearby marshy and wooded outskirts of the city, now long since developed. Some days Lawson rode around with his father in his buggy as he made house calls and observed the doctor at work. He admired his father immensely and I do not know of a time when Lawson did not want to be a doctor, too. It is frustrating to know so little about my grandfather’s life and education, as he was well read and imparted much of this enthusiasm to his son. As my Aunt Emilie wrote in 1963 to Dr. Alfred Bongiovanni, one of Lawson’s fellows, their father had “a vivid imagination and a great sense of humor, effervescent and kindly. He also liked colorful language. Characters from literature, especially Dickens and Thackeray, became alive in our household. When he felt the need of some strong expletive which would be frowned upon by his wife, he would quickly couple his remarks with a so-called Shakespearean quotation. He also had some lesser but equally colorful expressions. He enjoyed driving the horse and buggy used professionally, or the ‘trap’ used for family rides, with squirming children in the rear. He drove with a flourish, whether on a cobblestoned street or up Broadway, with its flowering park, overhanging trees, and fountains, to the country just beyond North Avenue. However, dad not only felt he should have absolute right-of-way but loudly called the wrath of Shakespeare with a few additions of his own upon anyone who chose to be upon the street”[3]. Lawson reported that his father addressed the offenders as “wild and woolly elephants” or “thou false Rodrigo villain, thou!”

The example of a father who had educated himself without apparent support from his family and made a prominent place for himself in the community was not lost on his son. The questioning mind which led to research was there. Also, Dr. Wilkins published a paper on the need to use a thermometer to measure a fever accurately, as opposed to feeling the patient’s brow and estimating the temperature as was then the custom.

On October 1, 1870, when my grandfather was barely out of medical school, the following article appeared in the Baltimore Sun [2]:

Cancer from Smoking—In the early part of this week Dr. G. Lawson Wilkins, assisted by Charles McCormick, a medical student, operated for the removal of a cancer of the lower lip on Mr. James Ewings of Canton Avenue. Mr. Ewings, who is an inveterate smoker, first noticed the appearance of the disease on the right side of the lower lip (the side he usually smokes on) over a year ago.

The other factor which enlivened Lawson’s life and contributed to his success was his education. Not satisfied with the public schools he saw in East Baltimore, my grandfather persuaded some of his neighbors to join him in hiring a carriage to take their children across town to the Baltimore Friends School which had recently moved into fine stone buildings in the 1700 block of Park Avenue with some of the best facilities in Baltimore—state of the art science laboratories, art rooms, library, gym, showers and, very soon, a swimming pool.

Lawson and Emilie both went to Friends School from kindergarten through twelfth grade, Lawson skipping two grades in early elementary school although he still said “grandmuvver” for “grandmother.” Both felt it was the best schooling they could have had and the gentle, principled atmosphere of Quaker education was a solace to these motherless children. While my father never subscribed to the Quaker philosophy, my experience with Quaker education leads me to believe that it had a lifelong influence on him. Baltimore had had a substantial Quaker community since 1656 and, to provide a “guarded education” for their children, a Friends school had been founded in 1784—the first school in the city, forty-five years before the first public school. The Quakers stressed equality of all people, believing “There is that of God in every man,” as well as simplicity in all things and non-violence. They emphasized perfectionism in all aspects of life and as a goal for society. The school had always accepted outsiders as well as Quakers and the students were generally children of professional people and prosperous merchants, college bound, but not the social elite of the city [4]. Lawson’s elementary education included French, German, painting, drawing, calisthenics, instrumental music, and voice culture! Heaven only knows how that contributed to his later singing ability.

## The Baltimore fire

At 10:48 a.m. on Sunday, the seventh of February 1904, the Baltimore Fire Department received an automatic alarm of a fire at a business in the heart of downtown Baltimore. By noon Baltimore was experiencing a devastating fire which lasted thirty hours and destroyed seventy square blocks. 1500 buildings, including many of the major banks and businesses, were reduced to ashes.

At home the neighborhood was showered with sparks and for hours my father’s family expected to be forced from their house. All night, a terrified Aunt Clara kept Lawson, aged nine, and Emilie, six, at the third floor window, praying. Finally the wind shifted and they could see that their prayers were answered: the house was out of danger.

**Figure 5:**
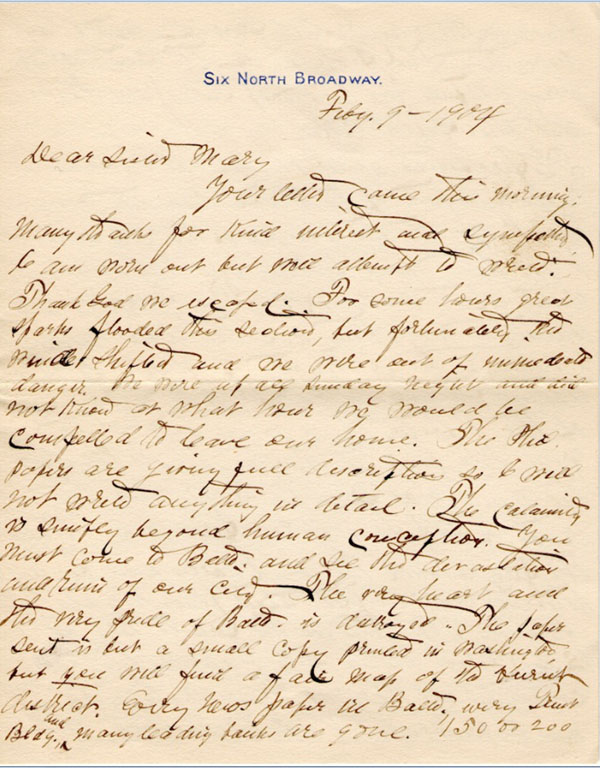
Letter by George L. Wilkins

**Figure 6:**
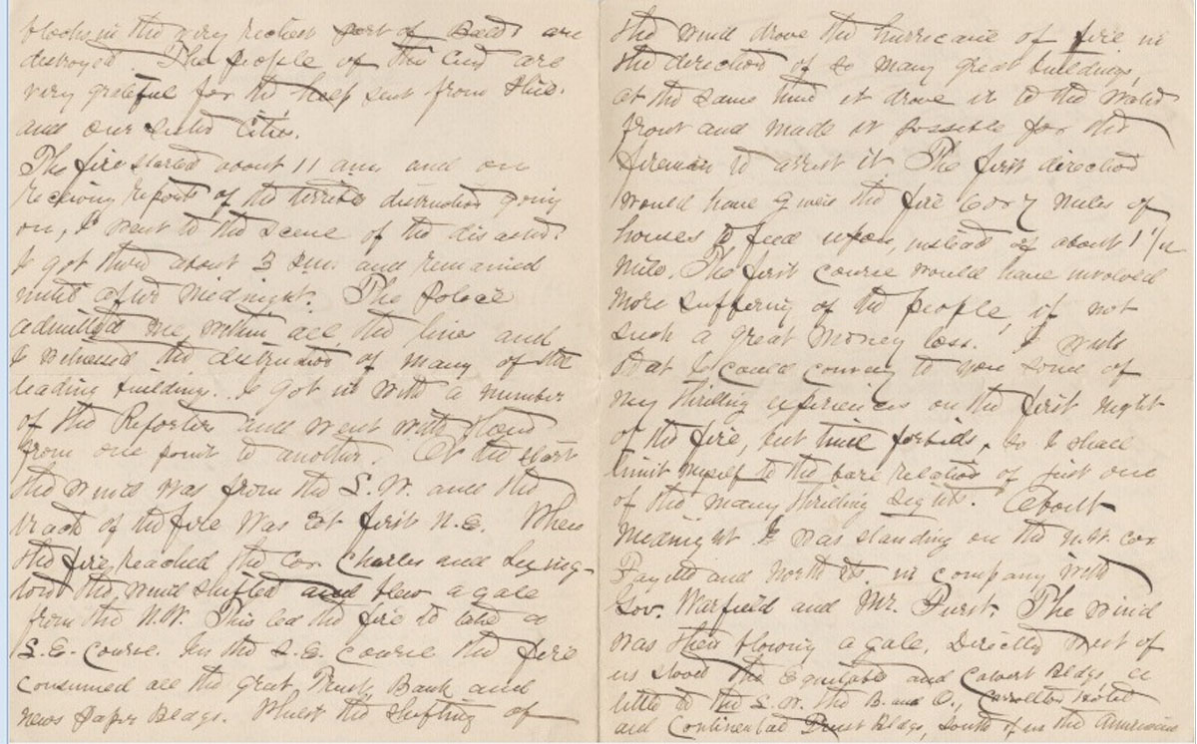
Letter by George L. Wilkins

**Figure 7:**
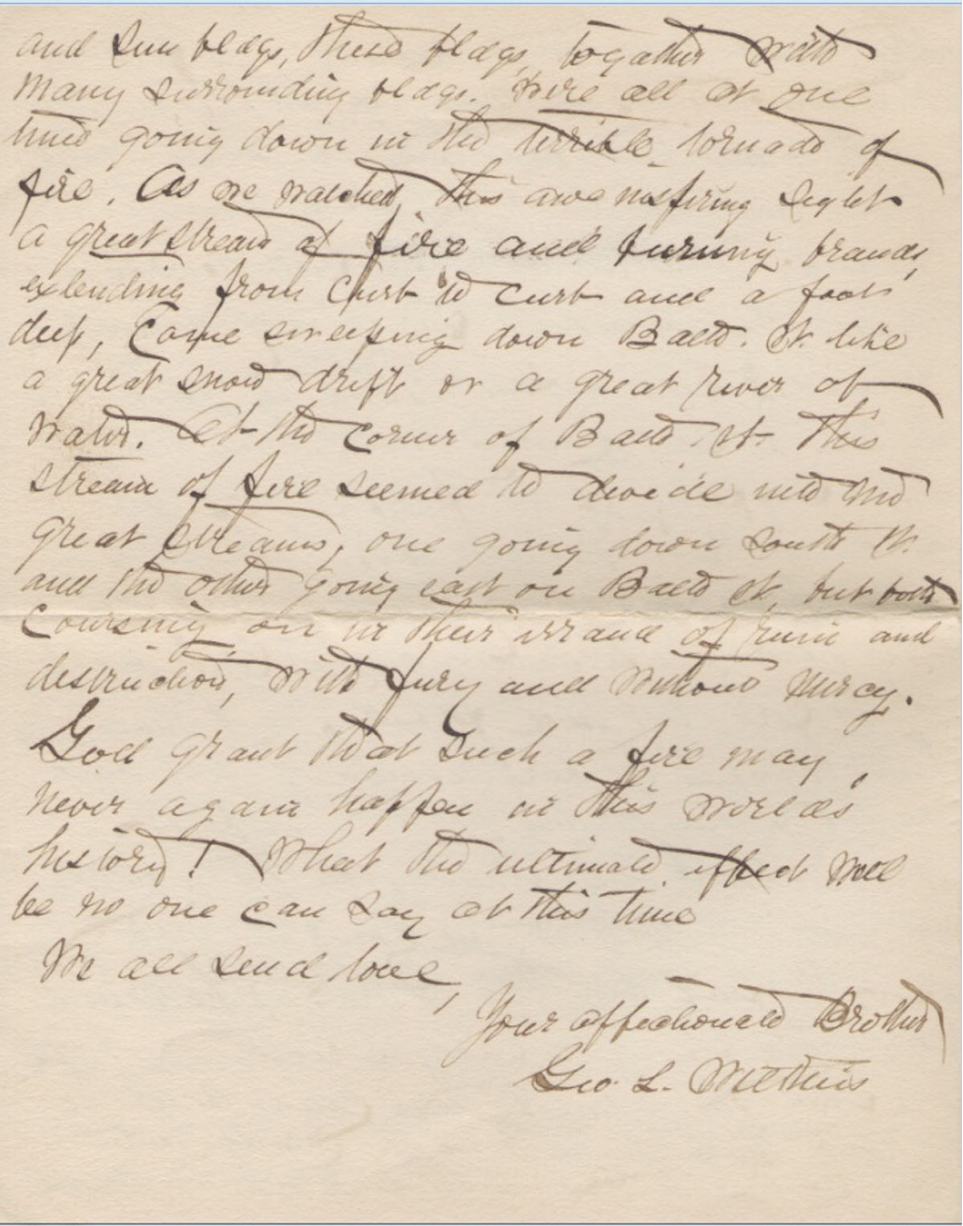
Letter by George L. Wilkins

Meanwhile, my grandfather was having an even more exciting time. I have a letter, dated two days later, from him to “Sister Mary”, (who was most likely Hattie’s half-sister with whom they had some communication) with a vivid description of the fire (Figures 5, 6, 7). His impulsive desire to be in the center of an historic event sounds like the sort of response Lawson might have made under similar circumstances. Upon receiving word of the conflagration downtown, he ran about a mile and a quarter to the scene and, joining a group of reporters, followed the fire at very close range as the winds swept it through the heart of the business district. As physician at the city jail since 1900, he was well known to the police. He was granted access to the front line and at times stood watching with the governor and other dignitaries.

## The stepmother

In 1905, the Wilkins household noticed that Dr. Wilkins made many mysterious telephone calls to Philadelphia—an unusual thing for that era. In April, Dr. Wilkins’ friends and patients were surprised to learn that he had married his dear friend, Annie B. Beaton, of Philadelphia and set out for Europe on a wedding trip. Aunt Clara probably suspected the romance—she had filled Lawson’s and Emilie’s ears with stories of wicked stepmothers. Perhaps she hoped to marry her sister’s widower instead.

A shy woman of thirty-eight with little experience with children other than the Sunday school classes she had taught, Annie seemed ill-equipped to be a stepmother. My father and Aunt Emilie said they never felt any warmth from her. The daughter of Scots, she was a strict Presbyterian with staid ways. In defiance, Lawson and Emilie would run home from church on Sunday mornings to play one record on the gramophone before Annie got there. My grandfather took to keeping his bottle of whisky in his galoshes in the hall closet and tried to control his explosive outbursts and humor. She and my grandfather had a marriage of thirty years and my father and aunt were dutiful children but I always felt a tension between her and us.

My father seemed to excel at all he did in school. He learned French, German and Latin and pulled out his notebooks of impeccable translations of Caesar, Cicero and Virgil when I was studying the same material more than forty years later, not to provide help (it must be all my own work) but to enjoy recalling his classical education. He loved the sciences and retained long passages of the literature he had read all his life. Although slight of build, about 110-115 pounds, he played on the basketball and lacrosse teams. He held various class offices and edited the school newspaper and yearbook. He was popular and made many lifelong friends, including Felix Morley, an author and editor, later president of Haverford College, and a number of children of the successful German Jewish merchants who were making important places for themselves in Baltimore [5].

Emilie felt she had a sadder childhood than Lawson and resented his greater freedom. She, too, appreciated what Friends School offered and made lifelong friends there but was more tied to home. She felt her father wanted her to sit at his knee and let him stroke her golden curls, like a perfect Victorian daughter. She had to fight to go to college, move away from home and create a career.

My grandfather did not neglect Lawson’s and Emilie’s musical education, either. One of his patients was Austin Conradi, the first violinist in the Baltimore Symphony Orchestra. In lieu of paying his medical bills, my grandfather asked Mr. Conradi to teach Lawson the violin and Emilie the piano. After a year or so, Mr. Conradi beseeched my grandfather to let him go back to paying his bills and to be relieved of teaching Lawson and Emilie. Despite this diagnosed lack of musical ability, my father, in his perfectionist way, had mastered the fingering and bowing of the violin. Thirty or so years later, when I undertook to learn the viola, my father picked it up and was able to play to the extent that my mother bought him a secondhand violin and, with her good piano playing holding us together, we happily made some excruciating music together.

Lawson’s and Emilie’s singing was notorious, also, but whereas Emilie avoided occasions when she might have to sing, Lawson loved to join in or lead with his extraordinarily deep bass whenever a singing party could be stirred up. In adult life he enjoyed the symphony and opera, too.

## High school and college

I have much less information about Lawson’s high school and college years than about his early life. He completed high school in three years by taking extra courses each year. There was apparently no question of his going away to college and he was proud and happy to have gone to Johns Hopkins University. He made many friends, joined Phi Gamma Delta fraternity, may have played lacrosse and continued to excel at academic endeavors. In my childhood, he would tell funny stories about lab experiments and would hold forth, reciting Chaucer, Shakespeare and Milton at length at the dinner table. One summer he taught daily vacation Bible school (perhaps under pressure from his stepmother) and another year he waited tables at a resort in the Poconos. Such experiences were interspersed with tales of dances, picnics, canoe trips and mountain climbing. He graduated from college in 1914, Phi Beta Kappa.

The medical school curriculum Lawson studied was much longer, more rigorous and scientific than his father had. Johns Hopkins University had been founded in 1873, opened in 1876. With its emphasis on graduate schools and de-emphasis of the distinctions between graduate and undergraduate work, many considered it the first truly American university. Following a half century of efforts by the American Medical Association Committee on Medical Education and the Association of America Medical Colleges to raise the standards among medical schools, the founding of Johns Hopkins Medical School in 1893 marked the start of a new sort of medical education with higher standards. A four year college degree was required for entry; there was a four year curriculum; the school year lasted a full nine months; there were teaching laboratories and integrated college and hospital facilities to provide clinical training for advanced medical students. The Flexner Report of 1910, “Medical Education in the United States,” showed up the inadequacies of most medical schools, many of which closed while others improved their curricula; Johns Hopkins was the model.

Starting medical school in 1914, Lawson was in the best place at a very exciting time. William Osler had left Hopkins in 1905; I do not know which of the three other “greats,” William H. Welch, William S. Halsted or Howard A. Kelly, my father had contact with but he knew medical history was being made [6].

## World War I

Although the United States was still neutral early in 1917, there was a strong sentiment for sending an American Expeditionary Force to assist the Allies. Almost at the last moment, it was decided to include personnel for one base hospital. At first under the direction of the Red Cross, such medical units later came under control of the Army Medical Corps. Starting in May, Dr. Winford Smith, director of the Hopkins Hospital, rapidly developed plans to send staff, volunteers and material to create such a hospital. Within the month this had been put into effect. The concerns of the medical school faculty as to whether to accept third year medical students as volunteers were resolved by plans to provide the students with academic work overseas and to award their diplomas at the end of a year.

**Figure 8:**
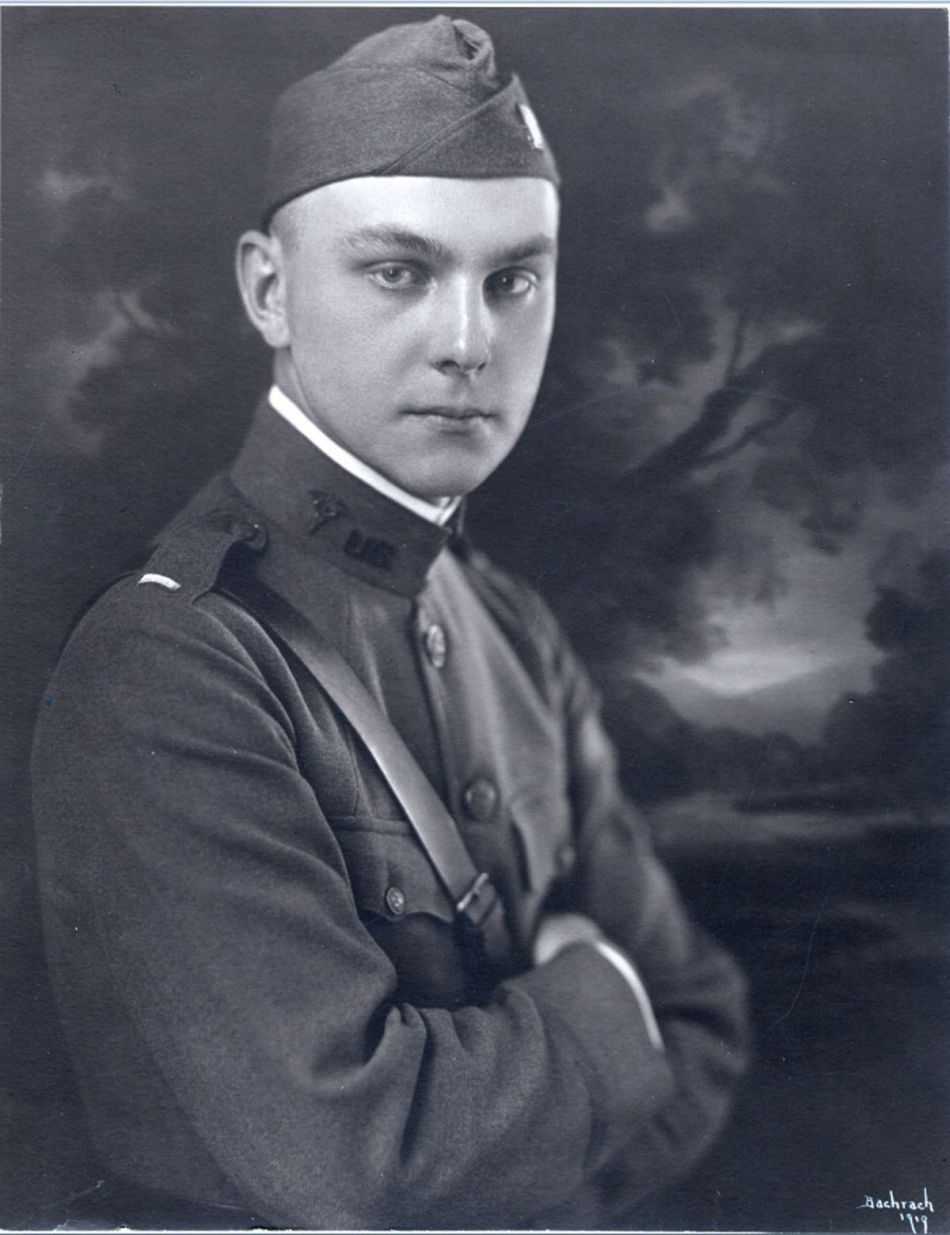
Lawson Wilkins in Johns Hopkins Medical Unit—1917

After a five day wait in New York, this hastily assembled crew of doctors, nurses, thirty-two medical students, cooks, stenographers, plumbers, carpenters, and so forth, led by Dr. J.M.T. Finney, set sail as part of a convoy on June 14, 1917 (Figure 8). Following a circuitous route to avoid submarines, they arrived in the port of St. Nazaire, France, on June 18. After a few days in the nearby town of Savenay, the unit traveled by train to Bazoilles, a small town in the Vosges Mountains in the east of France, arriving July 26. Taking over a 1000 bed temporary hospital, the members of the unit quickly prepared it for use. About eighty miles southeast of Reims, the hospital was near the battlegrounds of the Marne and Verdun. My father never told us about the wounded he saw but had colorful tales of the poilus, Senegalese, and other colonials he saw. He spoke of his distress at the death of two medical students and several nurses, who died of communicable diseases and their complications.

The students functioned as junior interns, anesthetists, assistants and laboratory technicians. They attended lecture courses by Johns Hopkins staff members and a course in Langres on the organization and administration of the Medical Corps, special problems of troop sanitation, the evacuation of the wounded and other responsibilities of the medical officers.

In April, 1918, Dr. Finney received a cable from the dean of the medical school announcing the graduation of all the medical students. As Lawson told it, the bugle blew for them to line up, Dr. Finney read the telegram granting them their degrees and then commanded them back to whatever they had been doing—Lawson had been cleaning bedpans. Later they were commissioned as first lieutenants.

Following the November 11, 1918 armistice, the Base 18 unit left Bazoilles on January 20, 1919. They sailed from St. Nazaire on February 1 and were discharged in New Jersey on February 20 [7].

While Lawson told his family little of the horrors of war, his nineteen months left him a lifelong Francophile. In his photograph album, in addition to snapshots of his comrades, the ships and hospital, there are many pictures of French villages, chateaux, soldiers, workmen and well-dressed civilians who sometimes entertained the visiting Americans. He especially loved the coast of Brittany and was thrilled to show me the places he had visited during his stay in St. Nazaire more than thirty years earlier.

## Early career

When Lawson returned from France, it was too late to obtain an internship at Johns Hopkins. He took an internship in internal medicine at Yale-New Haven Hospital—the only time in his career when he worked away from Baltimore.

The following year he accepted an internship in pediatrics at Hopkins. Still looking slim and boyish, by 1926 he grew a moustache to enhance his maturity and credibility. I know little about his early days of practice and work in the clinics at the Harriet Lane Home, the pediatric wing of the Johns Hopkins Hospital.

I do have evidence of his social life and good times in his photograph album. Like many of his fellow soldiers he stayed in the reserve or National Guard, attending camp at Tobyhanna, Pennsylvania, in the summers and spent time riding horseback. By 1925 he had acquired a sailboat, a 25-foot catboat with a 12-foot beam, into whose cockpit he crammed crowds of friends. How much he knew about sailing before that, I do not know but there were later tales of crazy cruises, groundings and surviving wild storms on Chesapeake Bay. Sailboats were a part of his life from then on.

In this era of Prohibition and despite his stepmother’s upbringing, liquor was always available for the good times, reportedly some bathtub gin. He combined his chemical expertise and his French experience to try to manufacture Grand Marnier, with some success—he claimed.

In addition to his old friends from school and medical school, he made many new friends, some parents of his pediatric patients, some in academia at Hopkins, on the staff of the Baltimore Sun and in various businesses. Some were from Goucher College, friends of his younger sister Emilie—and that may have been how he met Lucile Mahool.

## Marriage

I do not know how or when my mother and father met. Her photograph album contains a number of pictures of Lucile riding horseback in Howard County with a long-term beau and with a group of friends at Cape May, both in 1925. Lucile and Lawson were married June 9, 1926—(perhaps after a whirlwind romance?)

My father said that, because of his puritanical upbringing, he had determined to marry “the most worldly woman” he could find—if not a Catholic, an Episcopalian. They were married in what they described as the cellar of the Episcopal Cathedral of the Incarnation, (the undercroft), as construction of the cathedral was just beginning. There was a reception at the Mahools’ house in Roland Park, where there was some dissimulation about the punch, as the Mahools were teetotalers also, although not with the ferocity of Mrs. Wilkins’ beliefs. Perhaps Lawson’s father helped out.

My mother and father always seemed to adore each other and complemented each other remarkably. My mother, six years my father’s junior, came from families who had been in Baltimore and Baltimore County at least since the eighteenth century. Aside from an uncle who could not get elected to a second term as mayor of Baltimore because he was “too honest,” no one had done anything extraordinary. My maternal grandparents and their three children lived on 29^th^ Street with Aunt Bessie and Uncle Horatio, my grandmother’s siblings, until 1923 when, with Uncle Horatio’s help, they bought a lovely house at the top of Merrymount Road in Roland Park. Her family life seemed happy and secure, with numerous relatives around, trips to the mountains and beach, ice skating in the winter and tennis in the park. She had a brother four years younger and a sister eight years younger.

Lucile, called “Lu” by all her family and friends, had pretty, regular features, a radiant smile and fine brown hair which turned gray very young. Her ladylike demeanor masked a merry, at times wicked, sense of humor; everyone found her to be loads of fun and she was very popular. As my grandmother enjoyed bragging, “You know, she had eleven proposals—eleven DEFINITE proposals!”

Lucile graduated from Western High School, the academically oriented girls’ high school of the day, and Goucher College with a B.A. in French and Spanish. She played the piano quite well and loved gathering people around to sing. She was the first member of her family to go to college and the first to go abroad, taking a chaperoned tour of Europe in 1923. She taught kindergarten and first grade and was teaching at Baltimore Friends School when she got married.

The newly-weds had a honeymoon in Bermuda where Lawson distinguished himself by falling in the water in his white flannel trousers; when the rigging on their rented sailboat fouled, he immediately climbed the mast to fix it, not realizing this boat did not have the stability of his beamy old “Typhoon,” leaving Lucile shrieking and visualizing herself a widow.

They settled in the Calvert Court Apartments on Calvert Street where Lucile said she had a great deal to learn about cooking. In those days, and until World War II, she phoned Mr. Lissey at the Wyman Park Market every morning, as did her mother, to ask him what looked good and to have him send her order over. They had a cook until World War II and a laundress two days a week.

## Children

On September 23, 1927, George Lawson Wilkins, II, was born. He was always called “Skippy;” my father was the captain of the ship and his son was to be the skipper. He was a merry, high-spirited little boy.

The stock market crash of 1929 and the Depression caused belt-tightening and anxiety for my parents. Even if my father was pretty well-established as a practicing pediatrician, not all of his patients’ families were. When the banks closed, some people came to pay their bills in cash, thinking first of the doctor to whom they were grateful. Others provided produce from their farms or firewood. Few did not try to pay, except the Gypsy King, who skipped town after his daughter recovered from her pneumonia, for which my father had made daily visits.

My father sold his boat and gave up memberships in the Baltimore Country Club and the Gibson Island Club. He never joined again after the Depression; country clubs did not appeal to him. He and my mother were frugal and I always felt they had anxiety left from the Depression years.

I was born October 11, 1931, and named Elizabeth Biays after an ancestor of my mother’s. My family was living in an apartment in Roland Park in the home of Dr. and Mrs. Frank Ford who had become dear friends. We soon moved to a rented house a few blocks away where we lived until 1939 when my father bought the house on Edgevale Road where he lived until his death.

Roland Park, one of the first planned communities in the country, was designed by the firm of Frederick Olmstead in the 1890’s. The streets and footpaths followed the hilly contours of the land. There was a variety of housing, mostly shingle style or English cottage style, including imposing mansions, large comfortable houses, more modest homes, double houses and an apartment house, all designed by architects. Some enclosed lots were left empty for playgrounds and there were some large wooded areas. Many streets were lined with elms. I took all this for granted as a child but, as an adult, came to realize what an idyllic neighborhood it was for children.

## Lawson relaxing

I have many distinct memories of my early childhood but saw little of my father; reading of his involvement with specialty clinics in addition to his burgeoning practice, I understand why he usually came home after my bedtime, had to see patients on Saturdays and Sundays and slept late on Sundays. Spare time on weekends he usually spent working strenuously in his garden, no matter how hot the day. Some nights, when not studying, he worked into the wee hours on elaborate ship models. He often complained of severe headaches (migraine or stress?) which he treated with pyramidon, a very powerful pain-killer which is no longer available in this country. If home in the early evening, he usually took a brief nap on our hard Empire sofa and was then reinvigorated for serious work until one or two a.m., a habit my aunt has described from his school days.

Into this busy schedule, he managed to fit time to attend the visiting Philadelphia Orchestra and Metropolitan Opera with my mother, often arriving home in the nick of time to don his tuxedo and gobble a sandwich in the car. And then there were the parties, often impromptu, with singing around the loaned piano (with a cracked soundboard) tactfully accompanied by my musical mother and lubricated by plenty of drink. Favorites were from Gilbert and Sullivan, Negro spirituals, The Scottish Student’s Songbook, The Book of a Thousand Songs and, especially, rowdy sea chantries, all sung lustily with Lawson’s basso profundo, sometimes on-tune! Friends of all sorts were mixed in these gatherings and shy or unfamiliar foreign visitors were invariably swept into the musicale. (Never in my or my mother’s presence did he perform any of the reportedly ribald standards sung at the medical students’ Pithotomy Club.)

Other memorable evenings in my early childhood were devoted to our version of charades, not the silent miming of later years but elaborate, spontaneous, costumed performances of complicated or abstruse words. In the attic was a large box of costumes not only for the children to dress up in but for the adult games. I have a very early memory of the young Dr. George Thorn, (who later became the Professor of Medicine at Harvard Medical School,) waking me to locate my father’s army uniform for a charade in which he was a telegraph boy delivering bad news—rather frightening for a small, sleepy child who could not quite yet distinguish drama from reality. A cheerier charade involved a lady draped on our Empire sofa as Cleopatra while my father portrayed Caesar with a garbage can lid for a shield. As with the singing parties, sleep upstairs was impossible, anyway, so we children came down in our pajamas and were often incorporated into the drama.

My father really knew how to let down his hair and have a rip-roaring good time and his enthusiasm was impossible to resist.

Perhaps Lawson’s most relaxed and joyous times in pre-War days were cruising vacations on the Chesapeake Bay. After giving up the Typhoon in 1931, my parents and friends chartered larger sailboats for cruises. Beginning in 1934 they cruised each summer on the Richard J. Vetra, a fifty-two-foot Chesapeake Bay oyster workboat which had been converted into a sailing pleasure boat (I couldn’t describe it as a yacht) by his boon companion, Milton Offutt. Milt, a former Baltimore Sun reporter, was now a professor of history of science at City College of New York. He and his wife had a summer place on the Severn River. Milt was a brilliant, bitter, eccentric, opinionated and very funny man who had a great influence on my childhood. My parents spent two weeks each summer with him and his wife on the then 70-year old Vetra, exploring remote areas of the Chesapeake and its tributaries which were quite isolated in those days. Milt and my father would stay up very late, drinking, discussing science and philosophy and solving the problems of the world. Milt sang almost as well as my father and each summer my parents returned with new songs (in the fifties I discovered we were all folk-singers!) including some Milt had written over the winter to describe escapades of the previous summer, and highly embellished accounts of their adventures.

After the adults-only cruise (which included Milt’s daughter, eight years my senior), when we had been left in the care of our grandparents or another willing relative, the family would spend two weeks in a rented cottage on the Severn River. The thirty-mile trip from Baltimore was a wild journey. My father usually was visiting patients till the last minute when the four Wilkinses would pile into the cars (my mother had a 1929 Model A Ford with a rumble seat) the cook, with boxes of food for the vacation, the Irish setter and whatever assorted cats were currently family members. The latter usually escaped from the hat box in which they were traveling, along with assorted bugs and bees which flew in the open windshield, wreaking havoc among the passengers.

The summer cottages varied wildly. Before leaving home, my father gave us typhoid fever shots. In my earliest memories there was a cramped cottage with a pump for iron-flavored water, a frightening out-house and neighbors who constantly played one record, “Flat-Foot Floogie with a Floy, Floy.” Another summer we were in a naval officer’s mansion outside Annapolis. It ran the gamut, but we always had fun. We swam and floated for hours in inner tubes in the warm, brackish water. We crabbed and fished for perch, sunfish and the occasional striped bass off the dock. We dug clay from the beach and sculpted. We walked to nearby farms for corn and tomatoes and made sightseeing and shopping excursions to Annapolis.

My father had an eleven-foot dinghy, sister-ship to Milt’s, built for us and taught us to sail. As the smallest, my position in the crew was to sit in the bottom, shift my slight weight and dodge the boom. I came to love sailing much later. No matter how primitive the quarters, my parents always invited Baltimore friends to come for a picnic and cooling swim, often staying on for singing parties on the dock where the harmony sounded even better.

Dad devoted himself with his usual intensity to these activities with us. Among the most memorable were the wild rainy-day games of Demon Pounce, a loose form of multiple solitaire where everyone was playing all the time and playing on other people’s cards, too, with lots of pushing and shouting. In a quieter vein, my parents and grandparents taught us to play bridge.

Christmas Eve, Dad usually did not get home till the stockings had been hung and we were in bed. He would bring from the basement an 8 by 8 foot wooden platform he had built, cover it with fresh moss and set the Christmas tree on it. The tree was decorated with old ornaments from his childhood and some new ones. Then he and my mother went to work creating the Christmas garden. My grandmother’s doll house stood in one corner, a German manger scene in another. Through the garden ran the track of Skippy’s Lionel train, a mirror formed a pond and every year the collection of little English lead figures—farm animals, villagers, workmen—increased. When we descended on Christmas to see the lighted tree with the train circling it, we were enchanted. One Christmas “Santa” had a broken rib from falling off a ladder putting the angel on top of the tree. One year he had embellished the lovely eight room dollhouse that Grandfather Wilkins had built for me when I was three; Dad put in electric lights and nice woodwork, including stairs with tiny hand rails and newel posts. My mother had wallpapered and made little curtains and rugs.

Christmas gardens were a Baltimore tradition because of the large German population. In addition to visiting those of friends, my father took us to the fire stations around town where the firemen had created large and wonderful gardens for visitors to enjoy.

Another family outing was to the circus. When I was about five, we were taken to meet Jack Earl “the Texas Giant.” Dad had gone to the circus grounds early in the morning to ask Jack Earl for a urine specimen for his developing endocrine research. When he returned for the performance with Mother and us children, he was sorely disappointed that the specimen barely covered the bottom of the gallon jug he had optimistically left. He expected much more from a giant! On that trip he introduced me to many of the midgets and dwarves in the side show; looking into those wizened, prematurely aged faces was a shock to a five year old. The first introduction to my father’s interesting specialty made for good stories at kindergarten. (I did not tell my friends about the kittens whose thyroids he had experimentally removed so that they always looked like babies.)

## Family

**Figure 9:**
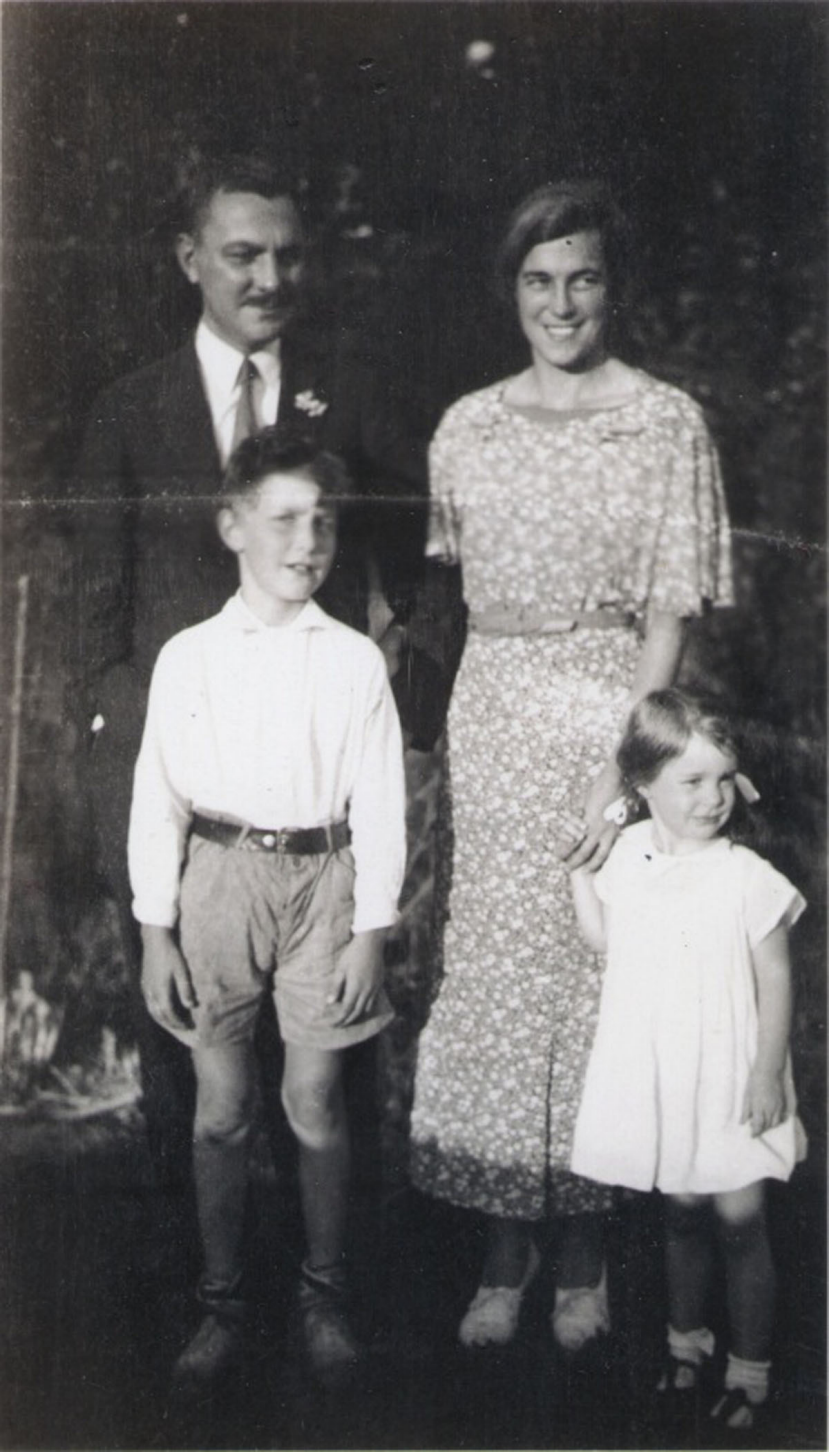
Wilkins family –1935

**Figure 10:**
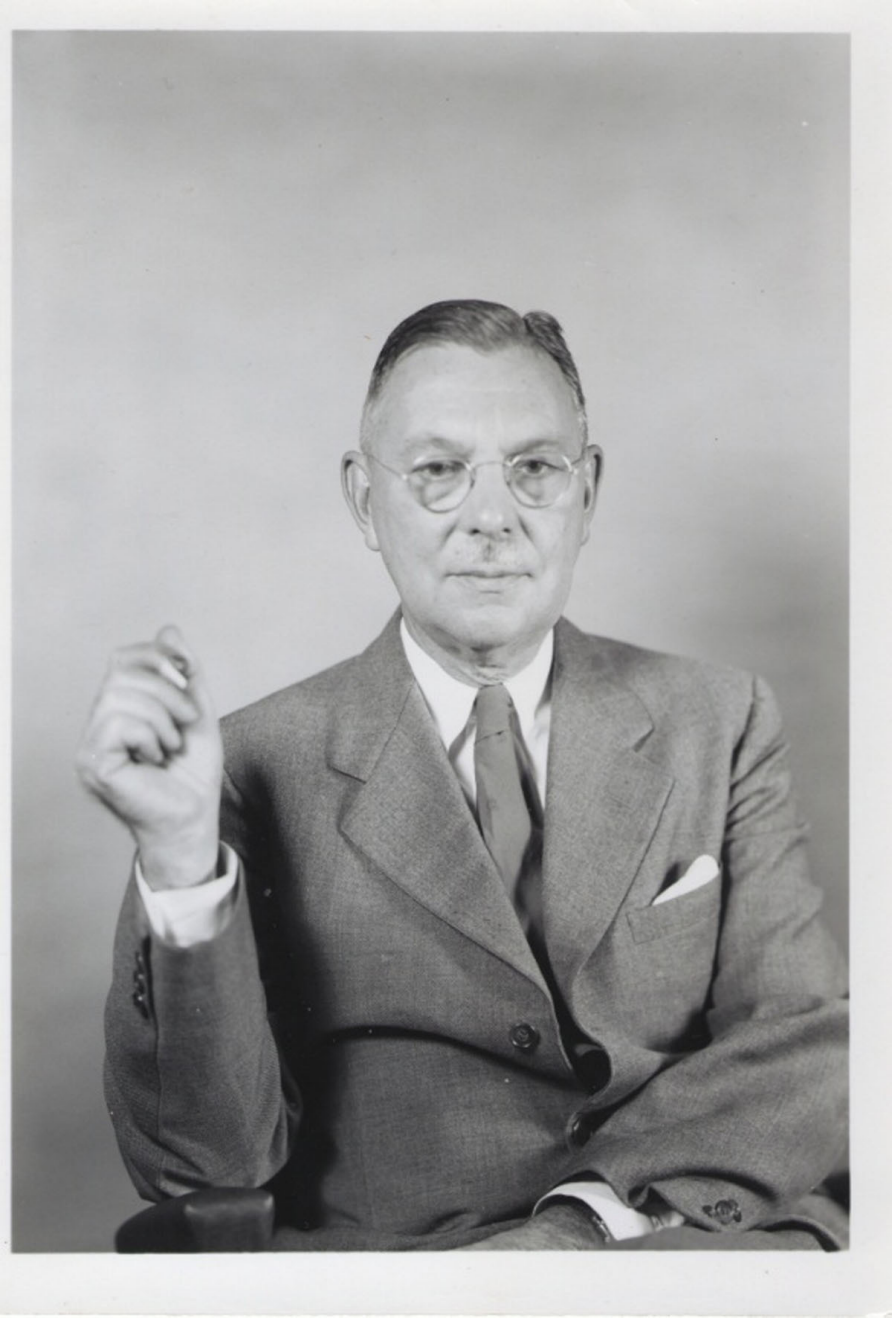
Lawson Wilkins –1938

It is obvious what a devoted father Lawson was but because he could not spend very much time with us, as a child I was much closer to my mother. She was wonderful at storytelling, singing, helping with dress-ups, puppets and plays, giving children’s parties with different themes and taking me to museums, concerts, plays, and so forth (Figures 9 &10). The other big influence in our house was Mary Johnson, the black cook, who worked for us from the time I was two until she left to work in a parachute factory in World War II. Having no children, she welcomed me into the kitchen where she taught me nursery rhymes, Negro spirituals, and lots of cooking. When my mother was out doing volunteer work for the YWCA, Community Chest, Johns Hopkins Hospital and other charities or playing bridge or attending the garden club, Mary and I had a glorious time in the kitchen.

Skippy, being four years older, seemed from another generation. He was very active with many friends playing cops and robbers, cowboys and Indians, football, and pranks where a little sister was a nuisance. I was much shyer, playing dolls, dressing up and such with one or two other little girls at a time.

Like his father before him, Lawson sent his children to the best private schools he could find, although the local public school was quite good. Skippy went to Roland Park Country School (RPCS) through fourth grade as it took boys as well as girls to accommodate families in those pre-two-car-family days. Then, with many classmates, he went on to the Gilman Country School. He did not thrive in its rigidly academic atmosphere and about seventh grade my parents transferred him to Baltimore Friends School which was more relaxed and understanding. He loved it. In adult life, I have wondered if Skip had a mild learning disability as he frequently had to be tutored; on the other hand, with his extroverted personality and many boyish interests, he may have just not seen the point in being too interested in his studies.

I went to RPCS from kindergarten through twelfth grade where, as a depression baby, I benefitted from very small classes; tuitions were extremely low as the private schools were struggling to stay open through the hard times and even gave discounts to families of doctors, teachers and clergy who were considered to be struggling on small salaries.

The Mahool grandparents lived nearby and we saw a great deal of them. My mother phoned her mother every day and my father remained devoted to his in-laws for life.

My Grandfather Wilkins had built a large house in the new development of Guilford in the 1920s. The sun-porch there was his office where he saw patients until about a week before his death at the age of 86. Dad took on some of his father’s very elderly patients and sometimes took me on his home visits to them; they doted on him and enjoyed seeing the beloved doctor’s four-year-old granddaughter.

My Grandmother Wilkins stayed on in the house on 39^th.^ Street, cared for by a faithful housekeeper till her death in 1945. My father managed her affairs and was always a responsible son. We saw a good deal of her.

My father’s sister married and lived in Pennsylvania; her family would visit Baltimore about once a year. My mother’s only sister, married and lived in Pennsylvania also, visited frequently, with her two sons who were considerably younger than me. Uncle Tom, my mother’s bachelor brother, lived a few blocks away with my grandparents.

## World War II

Our parents kept us aware of current events and in elementary school I knew that bad things were happening in Europe. When I was quite small I recall my parents allowed me to hear a speech by Hitler on the short-wave radio, explaining what an evil man he was.

In 1938 Dr. Walter Fleischmann, a Viennese physiologist, came to work in my father’s laboratory. Although I remember him and his family well, I never knew much about him or how he came to Baltimore. This spring (2013) after a hiatus of more than fifty years, I have been in touch with his daughter, Ruth Fleischmann Weiner, who has kindly provided me with information about Dr. Fleischmann and his family, from what her father told her.

“Walter was in Chicago at some sort of international medical meeting in the spring of 1938 and was advised by colleagues there to get his family out as soon as possible. I believe Lawson Wilkins offered Walter an appointment at Hopkins at that time. At any rate, he stayed in the U.S. and got visas for my mother and me and we sailed on the Westernland from Antwerp to New York in October of 1938—a month before my fourth birthday—and went by train to Baltimore. (I still have my green card, by the way.) In effect, Lawson Wilkins saved the lives of the Fleischmann family.

“Walter had quite a respectable publications list from his research in Vienna—a Festschrift was done for him in 1970 and I have many of the German language publications......Walter’s parents and siblings” (a brother and two sisters) “and their families emigrated at that time to England.” (Ruth informed me that Walter’s father, a physician in Vienna, had been a good friend of Sigmund Freud and had delivered his daughter, Anna.) “My mother, Gertrude, tried to bring her parents out but thanks to the U.S. State Department she could not get visas for them. In 1942 they were deported to Riga, shot by the Nazis, and lie in a mass grave somewhere in Latvia. Neither Walter nor Gertrude ever knew what happened to them; I only learned a couple of years ago in a letter from the Austrian archives.

“My half-brother Wolfgang Bernard (who went by Bernard in the U.S.) was living with his mother in France and was brought out by the American Unitarian Fellowship in 1940, along with 15 other Jewish children who had relatives in the U.S. Walter got Bernie’s mother out, as well as some more distant cousins. Bernie lived with us: his mother lived in New York and eventually went back to Vienna. Bernie died in 1987, very suddenly of heart disease; his mother actually outlived him by a year or so. My mother died in 1947 of breast cancer that had been in remission.

“I started school at Baltimore Friends School because I knew no English at all...I was fluent in both languages in a couple of months. We had very little money and I went to public school after third grade. Sometime after the war Walter got a position at Army Chemical center at Edgewood though he kept up his connection with the Harriet Lane Home. (Claude Migeon would have a better sense of the time line than I.) Actually Walter put me to work in the Harriet Lane when I was still in high school, doing flame photometry. Eventually Walter became certified in pathology and was pathologist at several VA hospitals in Baltimore, North Carolina and Tennessee. He married Sisanne Kann (yet another Ph.D.) who continued to work with him. He worked and published until his diabetic retinopathy and atherosclerosis got bad enough that he could no longer work. He died in Johnson City, TN in 1979. Susanne Kann Fleischmann died in 1997.”

Ruth Fleischmann Weiner has a Ph. D. in chemistry and has had a very successful career. She lives in Albuquerque and works at Sandia National Laboratory. She has four daughters, is in touch with her relatives in England and has enjoyed revisiting Vienna [8].

Another Austrian refugee my parents helped to settle here was Dr. Walter Block, whose wife, Elsa, was a gentile, a cousin of the opera star, Lotte Lehmann. They had a small son, Peter. Again my parents provided hospitality until they were settled in. Dr. Block developed a medical practice, also, and stayed in Baltimore.

In September, 1939, our parents took us to the World’s Fair in New York. The B & O train ride, the hotel, the subway to Flushing and everything about the fair was thrilling to me but I was aware of a pervading sadness as my parents pointed out the closed pavilions of Czechoslovakia and Poland, and the worried atmosphere in the French, Dutch and Belgian displays.

When the war came, Lawson, in his late forties, was too old to enlist but I saw less of him than ever. He covered the practices of several other younger pediatricians, who were away in the service. He rose at seven when the phone would start ringing incessantly. Receiving forty or more calls during breakfast, he grew to hate the telephone and let forth a stream of oaths before answering each time. My mother was in terror that he would not stop swearing before picking up the phone but he always sounded calm and collected when he did. During my years with him, I heard a great deal of creative blasphemy and rage, but never sexual or scatological language. In between calls, he would mournfully address his breakfast: “Oh, little fried egg, shall I never be allowed to eat you warm?” Finally he adopted the practice of other doctors of billing a dollar for each phone consultation which may have reduced the volume of calls.

When he could leave home, sometimes having to dig the car out of the snow and put chains on the wheels, he was off to his hospital visits, numerous in those pre-antibiotic days, and home visits. In the afternoon he held office hours in the building he and Dr. Amos Koontz owned at 1014 St. Paul Street. He usually sent his secretary, the faithful Charlotte Childs, out for his lunch—a grilled cheese sandwich or package of cheese and peanut butter crackers and a chocolate milkshake—not very healthy fare to combine with his constant smoking. After office hours there would be more home visits, especially to children too sick to come out or families with no transportation, thanks to gasoline rationing. Racing from Dundalk to a farm family with eight children in Ellicott City to the hospitals and everywhere in between, he would arrive home for a belated dinner at nine or ten. He figured he sometimes drove eighty miles a day on his rounds; as a physician he had a large gas ration and priority for new cars if they were available. Late at night he would pursue his reading and writing on endocrinology, before having a stiff nightcap and turning in. Sometimes late at night I would hear the phone ring, my mother rouse him, his feet hit the floor and soon he would be off on another emergency call.

This could not have been an easy regimen for my mother but I never heard her complain. She had full responsibility for the house, (with Mary Johnson now working in a parachute factory) the children’s activities, and any social life there might be and always had a warm dinner and things running smoothly when he returned. She pitched in to Red Cross and hospital volunteer work.

**Figure 11:**
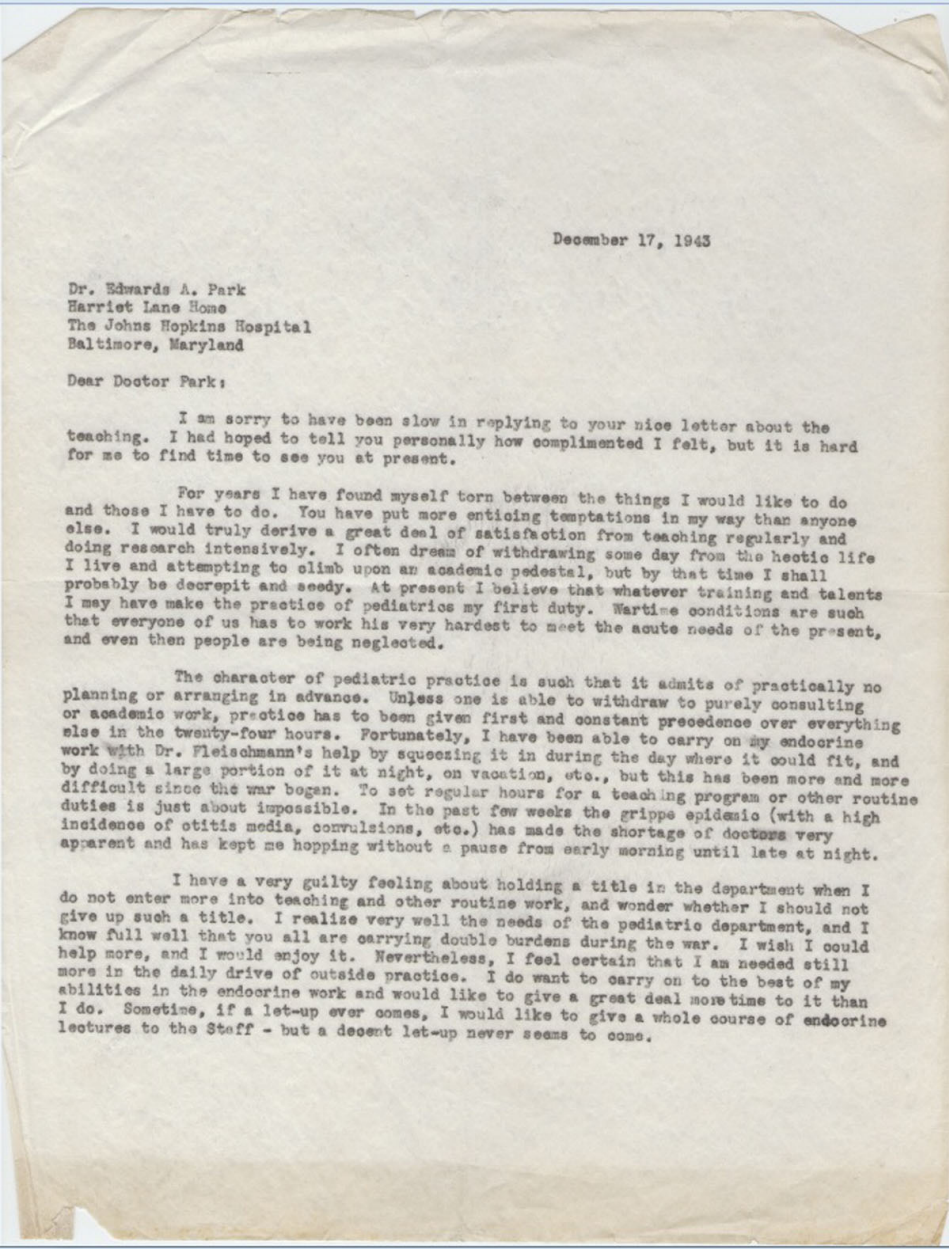
Letter to Dr. Park

**Figure 12:**
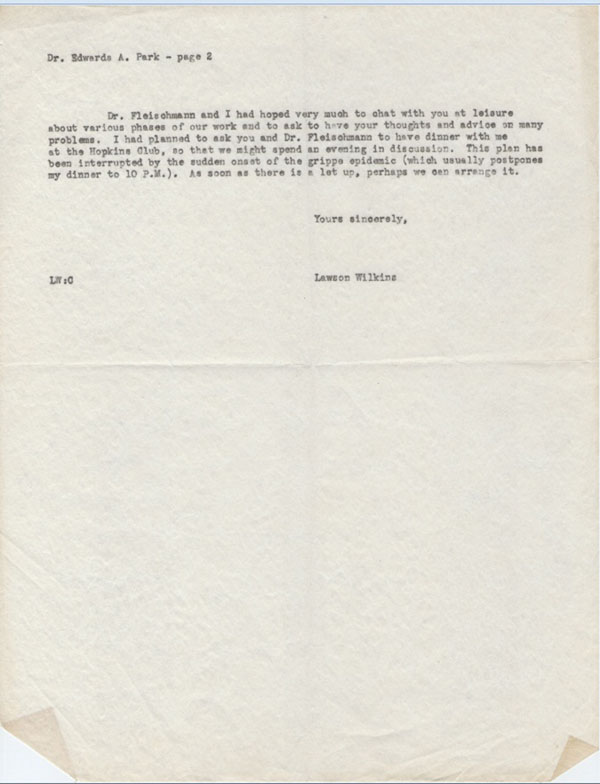
Letter to Dr. Park

In an interesting letter dated December 17, 1943, Lawson explained why he could not accept an offer from Dr. Edward Park, Chief of Pediatrics, to take an academic position in the Hopkins Medical School (Figures 11, 12).

## Skippy’s death

Saturday, January 15, 1944, was a dreary, sleety day. I was reading in bed, suffering from a sore throat, when I heard the front door slam. Then I heard voices making an unfamiliar sound. Were they laughing wildly? In a few minutes, dad came to my room and told me that Skippy, aged 16, had been killed in a crash. The shock of seeing him cry uncontrollably was horrendous and made me realize that my parents could not always protect me or make things right. Our lives were drastically changed from that day on.

**Figure 13:**
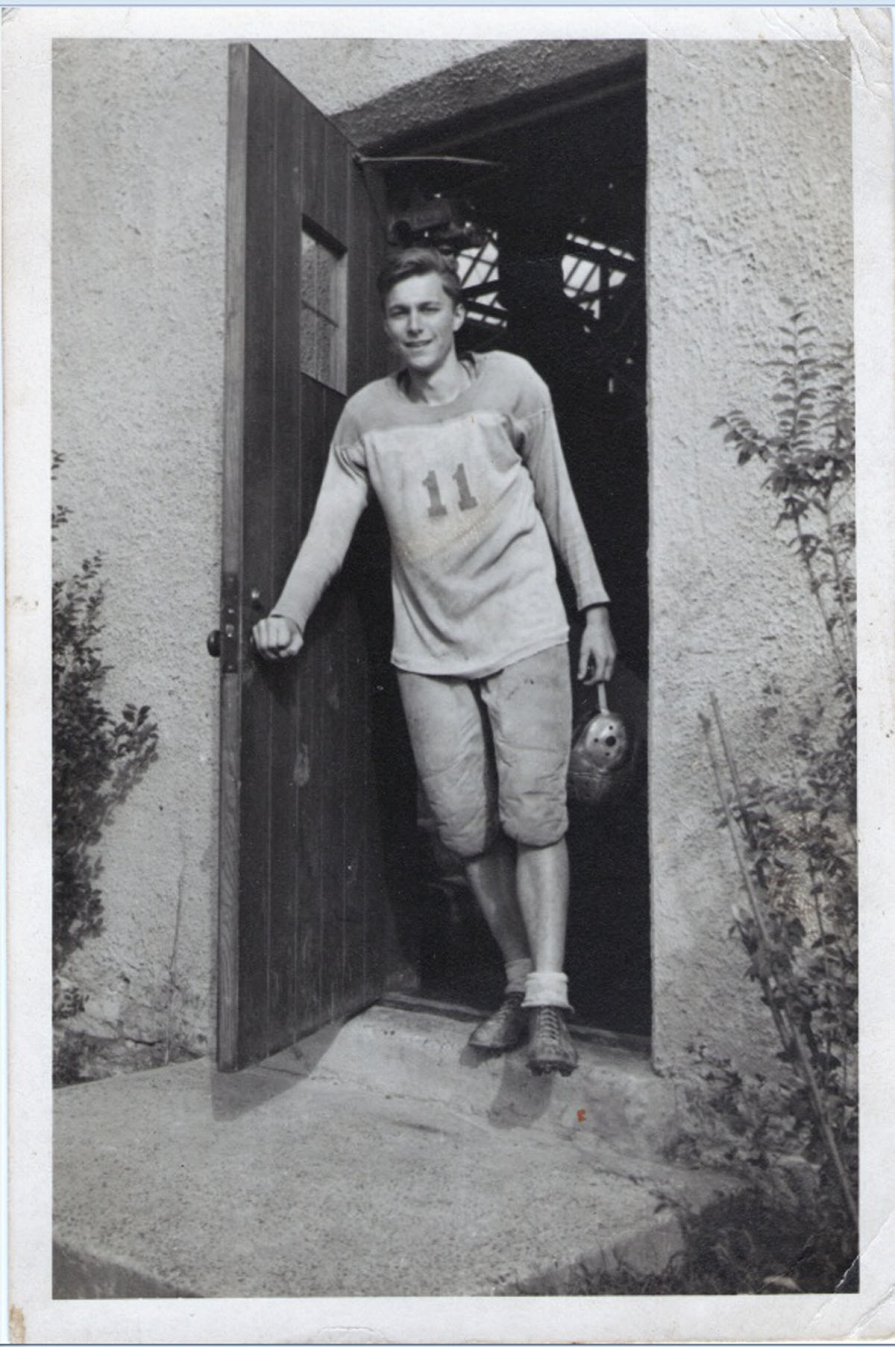
George Lawson Wilkins II—“Skippy”1927-1943

Because of the wartime shortage of laborers, Skip had had part-time and summer jobs since he was fourteen. He worked in the fields of a nursery. A tall, handsome, self-assured boy, he was popular and dated older girls who asked him to dances and parties because the older men were away in the army (Figure 13). He had a girlfriend two years older than he who was at college in Virginia. When he got his driver’s license in September 1943, he landed a job driving a mail truck for the post office. He was a junior at Friends, played varsity football and was vice president of the student council. He worked for the post office during the Christmas rush and was asked to stay on for weekends, leaving home before I was up. The old mail trucks were tall and top-heavy, resembling World War I ambulances. I never learned all the details of the accident but he struck a car, the truck turned over and he was dead on arrival at the emergency room of Union Memorial Hospital. My father who was seeing patients there was called to the emergency room to identify him. Then he had to drive home alone to tell my mother and me.

Because of wartime gas shortages, it was decided to hold Skip’s funeral at home, instead of a church or funeral home. He was laid out in the dining room and for days there were streams of friends and relatives, condoling with us, bringing special foods, scarce in wartime, and flowers. It was a terrible, devastated time. In retrospect, I realize this time of expressing and sharing grief was the best way my parents could have endured the loss. My father always said that it was my mother’s love and strength that made it possible for him to bear it. They were very wise in allowing me, aged twelve, to participate but sheltering me also.

Many family routines were changed. For several years we did not vacation on the Severn but went to friends in Rehoboth and Aunt Emilie’s cottage on Lake Memphremagog in Vermont and toured New England and Quebec. Christmas was spent at Aunt Emilie’s in Bethlehem, Pennsylvania, where the sounds of carols sung in the Moravian church in German (as he had learned many of them as a child) reduced my father to uncontrollable sobs.

My parents took up new activities. Lawson went back to carpentry learned in his childhood and redesigned and paneled our living room, replacing the Mission-style mantel piece with a graceful colonial one and building bookcases. He built a charming Sheraton style serving table. His gardening became even more strenuous, including designing and laying a flagstone terrace; the garden which had been like a cement parking lot when the previous owners (and their nine children) moved out, became a showplace, requiring trips to York, Pennsylvania, for special perennials. My mother acquired a fine Knabe grand piano from the estate of a very musical man and made more time to play. This was the era when, by chance, I took up the viola with the school orchestra, inspiring Lawson to renew his acquaintance with the violin. Any friend or neighbor who played an instrument was dragooned into joining our musicales. It was a good thing that our neighborhood had large yards where the sound could dissipate.

About two years after Skip’s death, Dr. Park’s successor, Dr. Francis Schwentker, offered my father a full-time position to carry out pediatric endocrinology. With the doctors back from the war, Lawson felt free to follow his passion for research. He also felt exhausted by the stressful war years and was willing to accept a lower income to go full-time. My mother, too, was delighted as she felt that pace would kill him and she was frequently very anxious when he was late coming home from his rounds of visits. Best of all, he was able to spend more time with her and me.

## Beginning to reap the rewards of his labors

My parents began to travel, attending medical meetings in other parts of the country and Canada.

I do not know who the first of Lawson’s fellows was. In 1947 or 1948, Salvador de Majo, who had left Argentina because of the Peron dictatorship, came to work with him. This quiet, gentle doctor was welcomed into family gatherings, subjected to the music, trips to the Severn and Maryland Hunt Cup, when my mother would polish her college Spanish; he remained a lifelong friend.

If Dad had any hopes of my heading into medicine (unlikely), they were not to come true. He took great pride in the fact that I did quite well at Roland Park Country School, getting good marks in the required biology and chemistry courses, but I never felt drawn to or thrilled by them. The sciences were the one weak area at the school and the way they were presented made them seem pretty incomprehensible to me. (The textbook for my introductory college biology course he also declared to be much too detailed and inappropriate.) If I had a strong drive to go into medicine I expect he would have supported me. He pointed out Harriet Guild, Helen Taussig, (bright lights at Hopkins), Mary Goodwin, Jean Stifler (practicing pediatricians), and Lydia Edwards, (researcher in mycobacteria who had an eminent career at NIH, UNRRHA and WHO) as wonderful role models, but always stressed what a hard profession it was for women. My mother steered me away from nursing with tales of bedpans and the servitude nursing students endured. Both parents encouraged me to volunteer at Hopkins Hospital, where I spent some high school summers running an antiquated cash register in the hectic Harriet Lane clinics, fetching charts and getting exposed to the variety of life there.

Lawson’s enthusiasm for his work entered all aspects of his life; he enjoyed explaining it to my mother and me at the dinner table. When I asked him in my early teens how to explain it to my schoolmates, he said: “Just tell them I’m a big sex man.” At thirteen I had bred a litter of Persian kittens to sell. Saturday night before an ad was to appear in the Baltimore Sun, he gathered Dr. Richard TeLinde, chief of gynecology and Dr. Frank Ford, chief of neurology, and other eminences around the table after dinner to determine the sex of the kittens. All four kittens were misdiagnosed, and when they were returned I had to refund the money paid for them; I did not earn enough to repay my parents the breeder’s fee.

When it came time for college, my parents would have liked to have me near home at Goucher but realized I was ready to stretch my wings farther. RPCS programmed as many students as possible to go to Wellesley, but, after thirteen years of sheltered female education, I chose Swarthmore. My father, who hated cold weather and felt somewhat depressed in winter, had dropped hints about the misery of New England weather and the dangers of winter sports (just look at Dr. TeLinde, who limped from a hip broken years before figure skating!) My parents were delighted to have me just outside Philadelphia, and were impressed with the academic atmosphere but, when they met some of the guys I went out with and realized how liberal the college was (this was the McCarthy era!), began to suggest that it would be nice if I would finish up at Goucher and meet more nice Baltimore boys. Considering their loss five years before, I have always appreciated that they resisted the urge to shelter me more than they did.

My parents usually planned a big party, with singing, around Christmas, when I was home from college, which included his fellows and house staff members he thought congenial. It was here I met Drs. John Crigler, David Smith, Alfred Bongiovanni, Gordon Kennedy, George Clayton, Jud Van Wyk, Mel Grumbach, and many others but do not recall who worked with Lawson in which years. Later I came to know practically all the fellows he had and was impressed with what a brilliant and lively group they were. He loved them all, as surrogate sons, and delighted in their successes. I could feel the love they reciprocated for him.

## 1950

**Figure 14:**
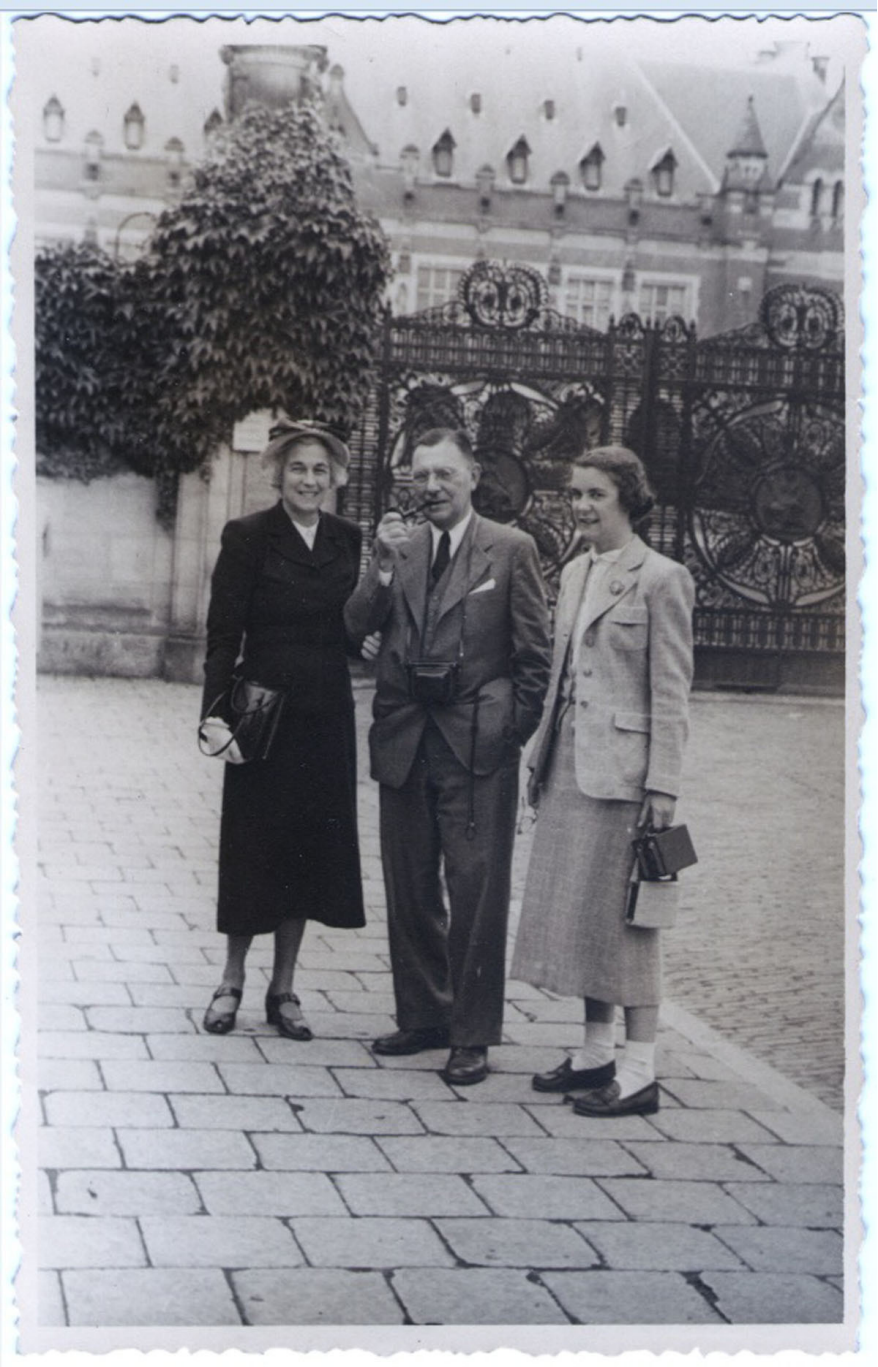
Lawson, Lucile and Betsy Wilkins, The Hague, 1950

One fellow whose arrival I recall vividly was Claude Migeon. Lawson had decided to attend the first International Congress of Pediatrics in Zurich in the summer of 1950. After the difficult events of the 1940s, with Europe in recovery and feeling a little more affluent than before, Lawson found this a good opportunity to take my mother and me to Europe. I had just finished my first year at college. He meticulously researched and planned every detail of the trip and treated himself to his first 35 millimeter camera. The trip included England, Wales, Holland, France, Switzerland and a bit of Germany (Figure 14).

Claude had been in touch with Lawson and came to meet with him at the Hotel de Paris. My mother and I met him in the lobby as we were on our way out. Meeting a Parisian (for the present), and recalling highlights of her 1923 visit to Paris, she sought his opinion on where to shop for kid gloves and perfume. I do not recall Claude’s answer but I remember his flabbergasted expression. Then my father asked if Claude thought it would be appropriate to take his eighteen-year-old daughter to the Follies Bergeres. Claude hesitated and then said, “Well, my grandmother took me when I was five.” I do not remember what we did with this information but we were all delighted when it was arranged for Claude to come to Baltimore, and he became a lifelong friend.

Another memorable highlight of the trip was our stay in Brittany which Dad had visited when he was stationed in St. Nazaire. We stayed in the ancient walled town of Vannes where his memories became ever more colorful, especially one of a picnic he went on with the daughters of a baker. He spied a bakery he was sure was the one and we trooped in. He told the middle-aged ladies who ran it that he was one of the young soldiers they had taken on a picnic at Chateau Suscinio thirty-three years before. He took many pictures of all of us smiling outside the bakery. When the pictures were developed back in Baltimore, revealing two rather plump ladies of unexceptional appearance, he said they had indeed aged—“But you should have seen their beautiful cousin who was visiting from Paris!” I don’t know what the ladies made of the experience.

The highlight of the trip was the 10-14 day stay in Zurich. I had never been to such a Congress. After the deprivations and isolation of war, the atmosphere was thrilling as this great number of scientists were able to meet one another and share their ideas. Lawson was very excited about the results he had had treating patients with cortisone, just recently available, and had a large display with many pictures and charts for the poster sessions. My mother and I had been enlisted in the early summer to help arrange the panels on the living room floor and packing and transporting them had been quite a job. The results exceeded Lawson’s wildest expectations as he became friendly with many international colleagues and was approached by the publisher Charles C. Thomas who asked him to write a textbook of Pediatric Endocrinology.

Back at home Lawson worked on the book with his usual intensity and it came out in record time; my mother and I spent hours with cards on the floor creating the index.

During subsequent summers of my college years, when I felt I should seek jobs to help with college expenses, Lawson found ways to direct my interests. One summer while my parents were on a professional trip, they arranged for me to share our house with John and Joan Hampson, psychiatrists he had brought onto his team to deal with the psychological aspects of patient care which he recognized were beyond his abilities. When I was planning to take an unexciting job in a secretarial pool, he inveigled a more interesting position for me as a case aide in the social service department at the hospital. (Although his sister had some frightful experiences in her career as a social worker in various cities, he felt this was definitely an acceptable profession for me). This summer job led to my accepting a job as a case aide at the hospital for a year after my graduation in 1953.

Living at home for a year after college and carpooling to work with my father was pleasant but I found the job limited and the atmosphere of Baltimore stifling. To continue in social work I must get a master’s degree and decided to go to Columbia, sharing an apartment in New York with my best friend from Baltimore. In the summer of 1954, before starting in school again, I took a three month trip in Europe with a college friend, Susan Harvey. Lawson was a visiting professor at Guys’ Hospital, London, and my parents were ensconced in a tiny, charming eighteenth century house. Susan and I made that the base for travels in England, sometimes with my parents who took us to nicer places than we could afford on our shoestring budgets. Susan and I had a grand time in Cambridge with Gordon and Minnie Kennedy and their children; Gordon had worked in Lawson’s department, sharing a miserable, hot apartment on Broadway with Claude. In Holland we visited Janny van Walbeek Kleyn, a hearty, fun-loving pediatric cardiologist whom we had met in 1950 when she worked with Dr. Helen Taussig, the renowned pediatric cardiologist who collaborated with Dr. Albert Blalock at Hopkins on the development of the operation to cure “blue babies.” In Copenhagen my parents joined us for a busy fun-filled week with Henning and Else Andersen. Henning, who had spent six months or so working with Lawson’s pediatric endocrine group, had taken a week’s vacation to entertain Lawson. Vacationing friends of theirs lent us their house. The Andersens’ sons were away at camp and Henning packed the week with merriment. When our energies would flag and we would suggest going back to the house to rest or sleep, he would decree that it was time to go to Tivoli; his favorite place was the Mysteriske Hus, a mirrored set of rooms where everything and everyone looked crazy. He would laugh so uproariously that we would become weak with laughter; the Danes certainly know how to enjoy life! In Copenhagen we also spent time with Lawson’s old friend, Lydia Edwards, now a pediatrician with WHO studying mycobacteria. In Paris we were entertained, also, by some of my father’s colleagues and had one memorable evening around a dinner table with one family, sharply divided over France’s policy in pulling out of Indo-China—a foretaste of what we were to experience with the U.S. policy in the sixties. After that, Susan and I went merrily off on our own through France, Germany and Italy, leaving my parents in London without word of us for six weeks, suffering alternately from rage and anxiety.

After getting my MSW I stayed on to work in an excellent social service department at the New York Hospital. I took a position working with previously undiagnosed tuberculosis patients with far advanced disease with lung cavitation. This was a very interesting group of patients, often with deep psychological problems, who had long hospitalizations while they were being treated experimentally with ioniazid. My father was very worried about me being exposed to TB and insisted that I should be inoculated with BCG. This had been used in Great Britain without any proof that it provided protection. As it turned one’s tuberculin test from negative to positive, the researchers with whom I worked strongly opposed my taking it and losing a means of learning if I contracted TB. My father was so worried and wanted to get any protection he could for me that I gave in and had the shot, to the annoyance of the “TB researchers.”

Those were exhilarating, fun-filled years. I relished using social work skills with patients struggling with illness, I enjoyed all that New York had to offer and felt I was meeting more interesting people in a week than in a year in Baltimore. I went home for holidays and my parents included me in parties and hoped I would find potential dates. They worried about me in the wilds of New York; each only visited me once in four years there, preferring not to think about the dangers of the city. Also, they were having a great time with travels connected with Dad’s increasing fame.

## Trip to South America

In November, 1957, Lawson was invited to an international conference in Buenos Aires, first class, all expenses paid for him and Lucile. Invitations also came from Brazil, Uruguay, Chile and Peru. For this once-in-a-lifetime trip, I persuaded my boss to let me take two years’ vacation back-to-back and my parents to let me join them; I contributed for my fares and some expenses. It was a fabulous month as we were lavishly entertained wherever we went. In Buenos Aires we renewed acquaintance with Dr. Salvador de Majo, who had left Argentina during the Peron dictatorship and worked with my father. We first met Dr. Cesar Bergada who later came to work with the endocrine group, bringing his lovely wife, Estela, six children and fabulous piano skills which enlivened many evenings. Dr. Jose Cara was an old friend in Cordoba who, like Salvador de Majo, had come to work with Lawson’s group during the worst of the Peron dictatorship. He and his wife, Maria, took us traveling in the Cordoba region. In every city, Lawson gave talks and early morning rounds before the sightseeing and entertainments began, ending with elegant dinners at late, Latin hours. In Lima we did not sit down to dinner until midnight, but still Dr. Nicanor Carmona picked Lawson up for rounds at seven a.m. the next morning. Fortunately, Lawson had been accustomed to such a schedule before and took advantage of the universal siesta.

## Betsy’s marriage

My parents had tried to mask their anxiety over whether and whom I might marry but they could not hide their delight when I became engaged to Philip McMaster. Although Phil had graduated from Hopkins Medical School and we had many mutual friends, we met not in Baltimore but at the New York Hospital where he was an intern. When we visited my parents on weekends, Lawson so enjoyed his company that he had to be reminded that Phil was here to see me and we might have plans to go out. When he very properly came to “ask for my hand” Lawson got so wrapped up in talking with him, that my mother and I had to linger in the kitchen for an hour, till we finally barged into the living room to remind them of the purpose of the visit.

Everything about Phil suited my parents, as well as me. He was now working at the NIH in immunology research, uncertain whether he would stay in research or go into practice. His father was a well-known scientist, one of the first members of the Rockefeller Institute. His family played the same kind of charades and sang the same kind of music as we did, as well as being lifelong sailors. His father even played the violin, self taught, but much more in key than Lawson.

**Figure 15:**
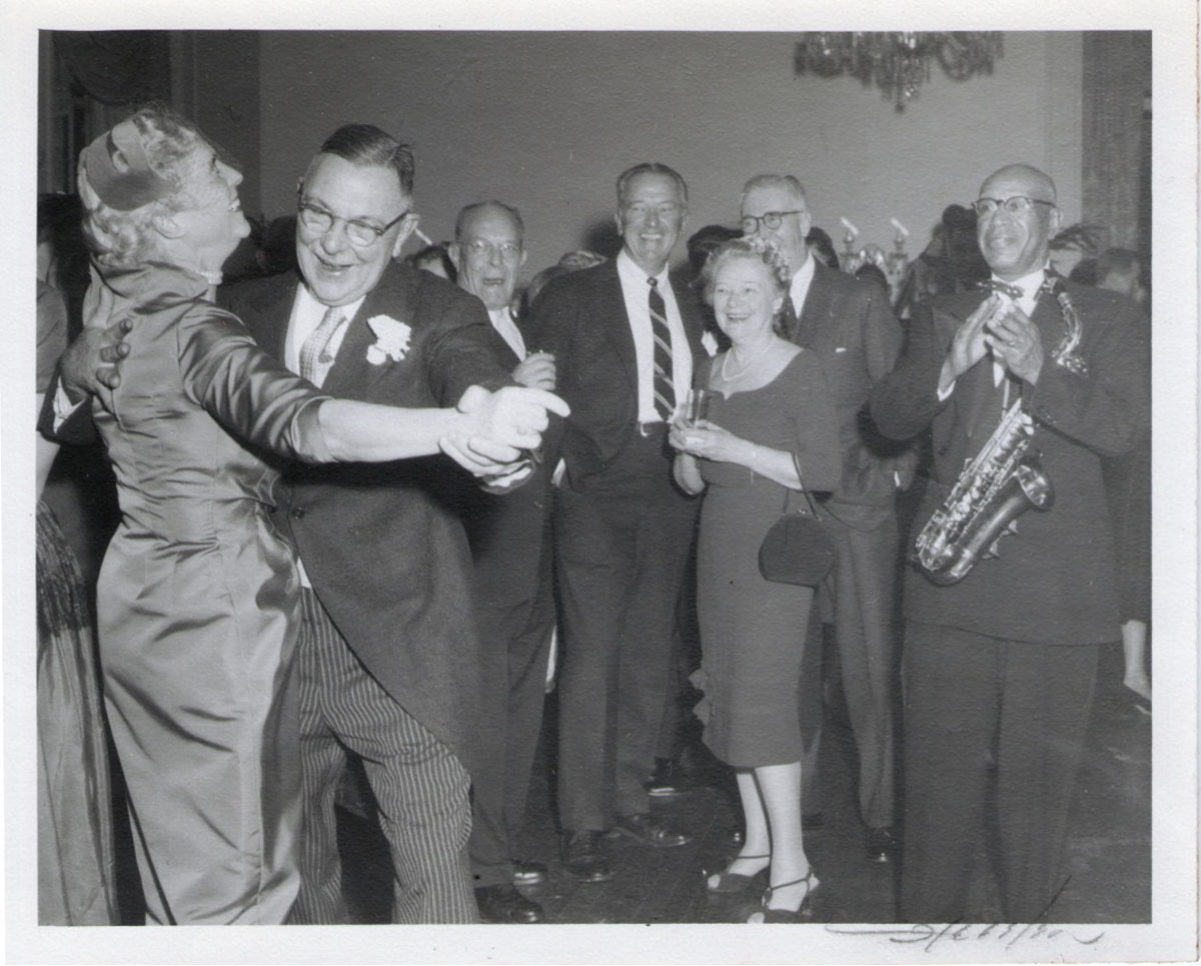
Wedding of Elizabeth Biays Wilkins and Dr. Philip Robert Bache McMaster December 13, 1958

**Figure 16:**
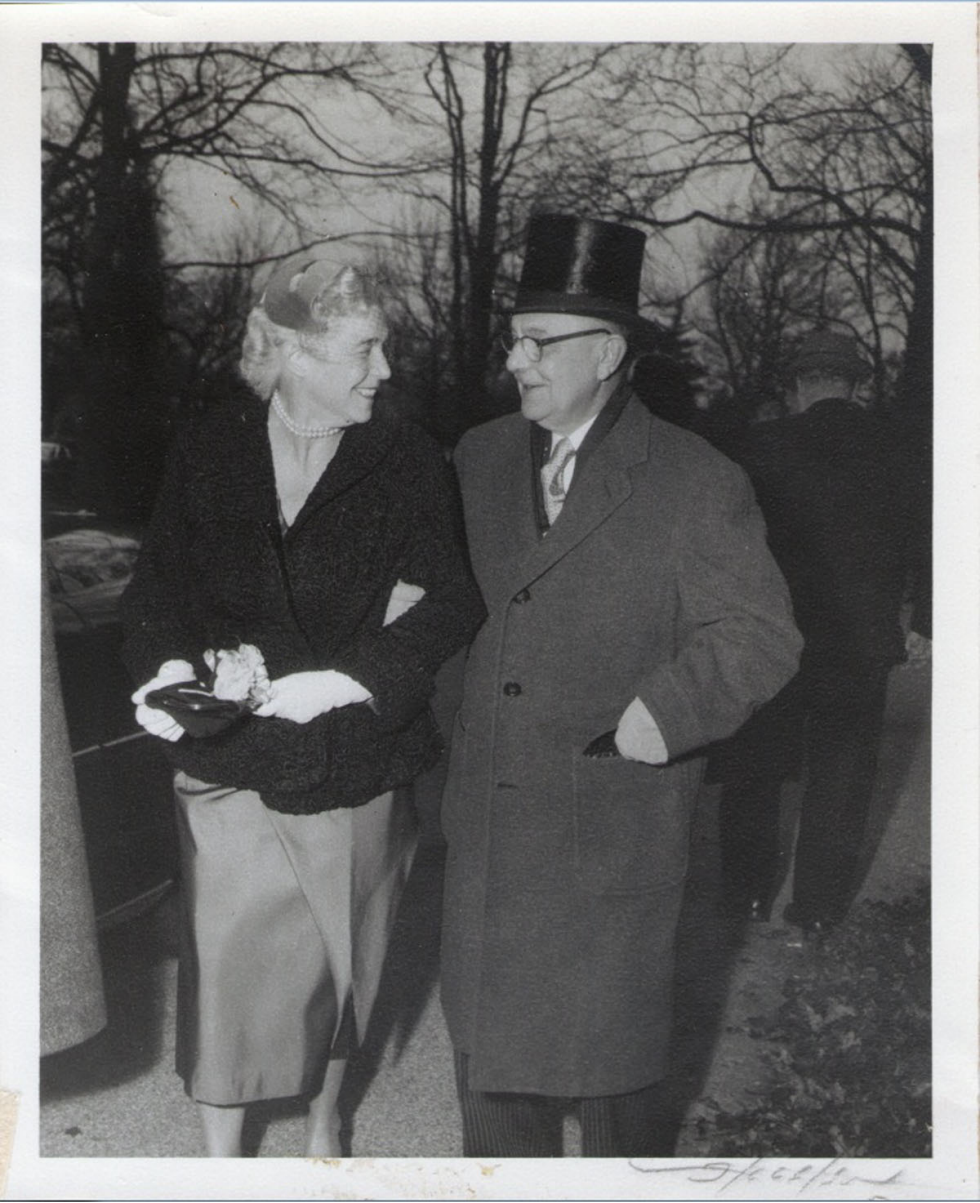
Wedding of Elizabeth Biays Wilkins and Dr. Philip Robert Bache McMaster December 13, 1958

**Figure 17:**
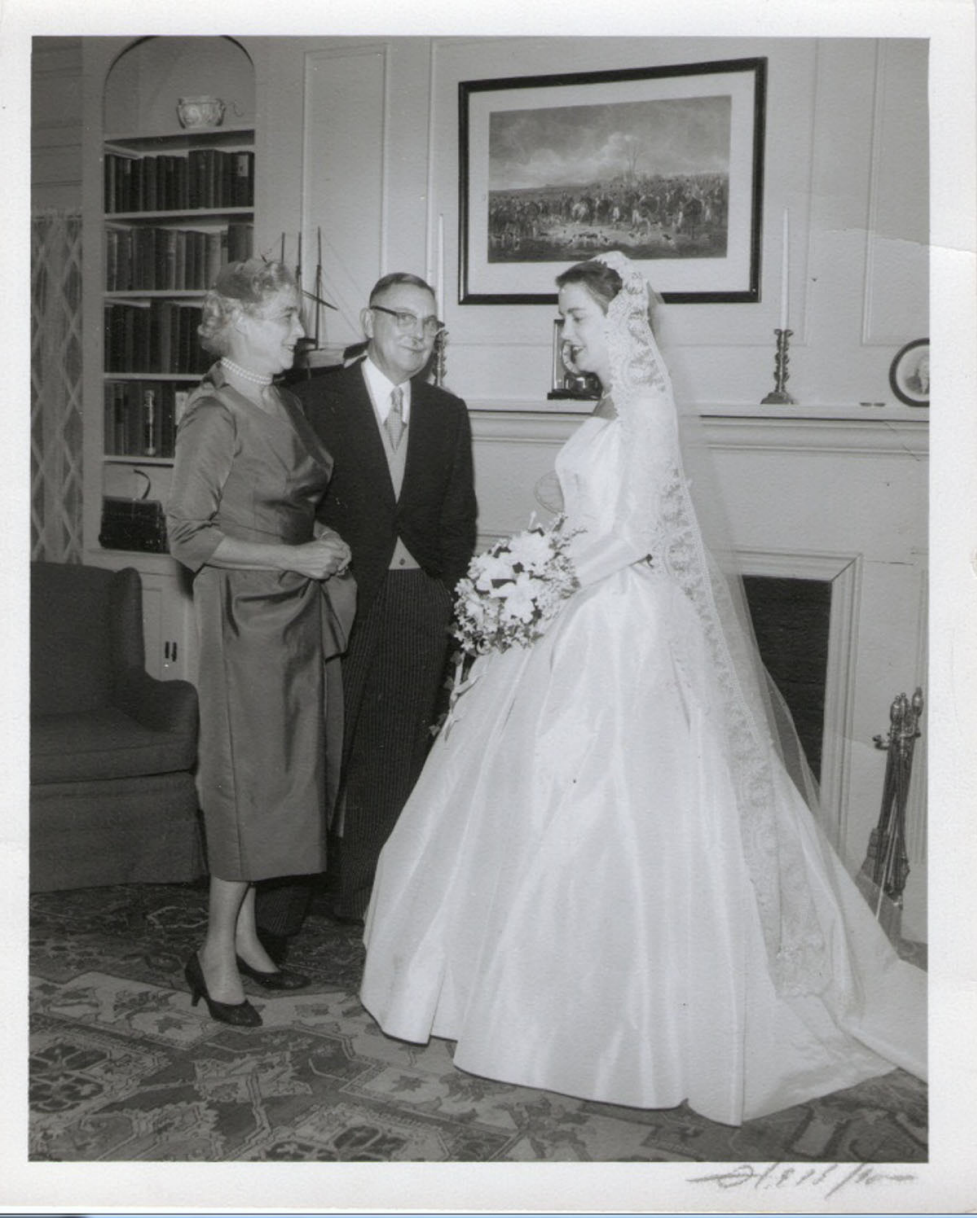
Wedding of Elizabeth Biays Wilkins and Dr. Philip Robert Bache McMaster December 13, 1958

**Figure 18:**
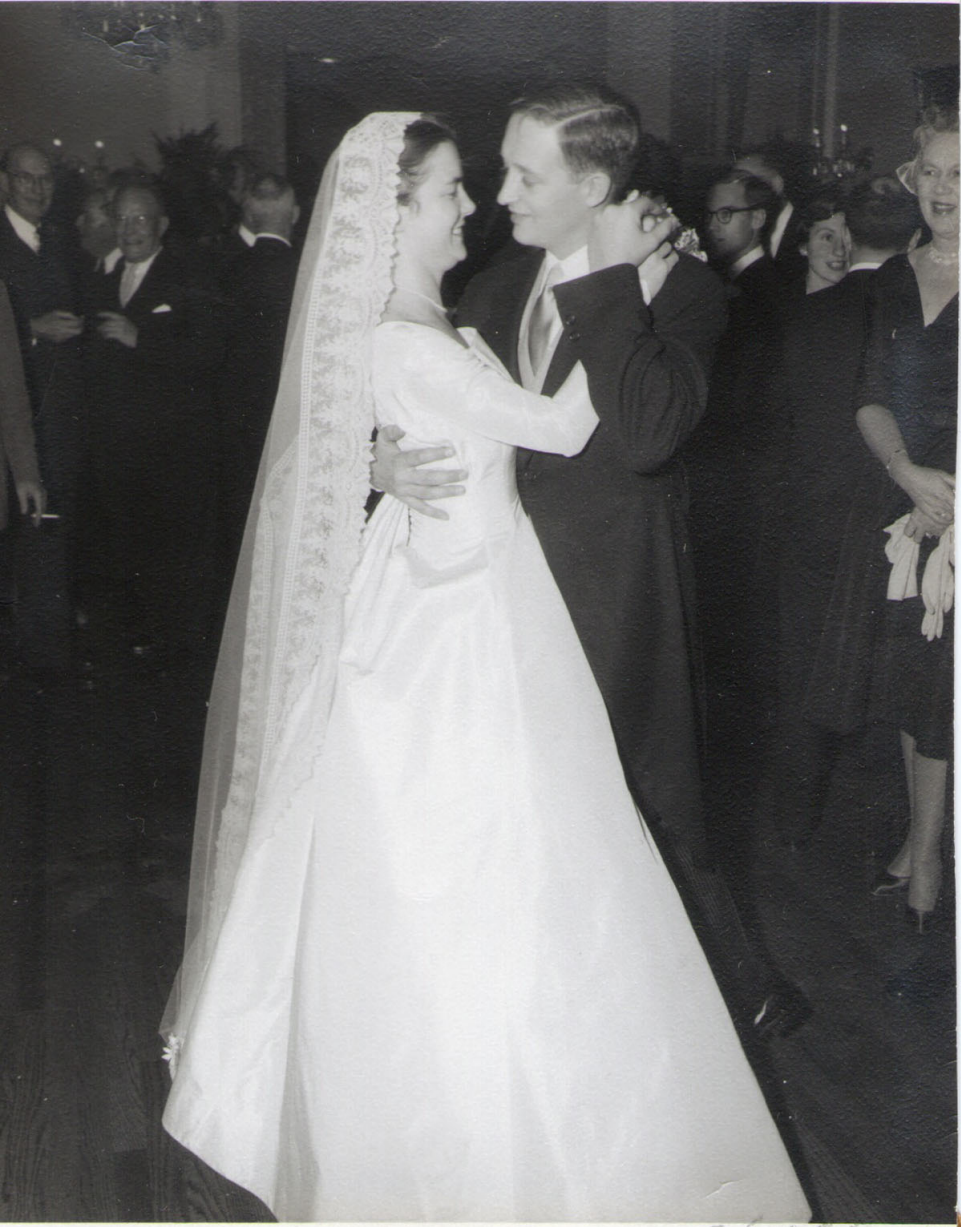
Wedding of Elizabeth Biays Wilkins and Dr. Philip Robert Bache McMaster December 13, 1958

My mother wanted to plan a June wedding but we insisted that we wanted to get married in December 1958. My parents pulled out all the stops and Lu had a great time planning the wedding and reception, a joyous occasion for us all. She looked radiantly beautiful in an electric blue silk faille dress (Figures 15, 16, 17, 18).

## Lucile’s death

Sometime in the first week of May, 1959, my father called me to say he was taking my mother to the hospital, as Lu had had a seizure and symptoms which he thought indicated a subdural hematoma. My heart sank with dread immediately and I strongly suspected the worst. Lawson remained in denial for a remarkably long time and could never bring himself to talk openly with Lu; they each kept up a pretense to protect the other. She died in a coma on June 10. Phil and I were so glad we had insisted on the earlier wedding date and given her so much happiness.

Even before Lu died, Lawson said he could never live alone; he would have to remarry. Following her death he was deeply despondent, having at least two auto crashes which took him to the hospital and exhausting some of his dearest friends with his outpouring of grief. As with Skip’s death, he personally wrote long responses to the hundreds of letters which poured in from all over the world. Not only had he lost Lu, but being sixty-five, he was required to step down from his position as head of the department. He was glad to turn it over to Drs. Migeon and Blizzard and would continue to work, write and lecture, but he felt doubly bereaved. I was torn between his constant neediness in Baltimore and getting settled in my own marriage and home in Bethesda.

## Remarriage

In September, 1960 Phil and I moved to Paris where he was to spend two years in research in immunochemistry at the Pasteur Institute. For me this was heaven and Lawson promised to visit us. Claude and Barbara Migeon, recently married, devoted immense amounts of time, energy and love to alleviating Lawson’s loneliness. They even let him come on their honeymoon, attending conferences in Scotland and Denmark together! After arriving in Paris, I received an international call (a rarity in those days) from Claude, informing me Lawson had had a coronary. He had told Claude to tell me not to come as he was in good hands and doing well. Very soon I received a letter in which he told me that he was engaged to Catrina Anderson Francis, called “Teence,” the sister of his lawyer, an old friend. They had a small wedding before a justice of the peace and visited us on their honeymoon in April, 1961. His happiness was infectious. We had a very merry time showing them our favorite haunts, introducing them to some of our friends and allowing ourselves to be taken to some fabulous restaurants. On a restricted diet following his coronary, Lawson would accept only two ladles full of rich sauce instead of three and was trying to stop smoking. He and Teence had some wonderful trips in the next two years.

## Lawson’s death

In the year following our return from France, Phil and I saw a great deal of Lawson and Teence; he was eagerly awaiting the birth of our first child. On September 27, 1963, Teence called us to come see him in the hospital; he had suffered another coronary. We were in time to tell him how much we loved him. He was very weak and tired, kept alive only by extreme measures. He died that day. Our son Charles was born November 2. Over the years I have come to recognize more and more how blessed I was to have had such a brilliant, original, highly principled, generous, loving, and funny father, and a lovely mother who, with her warmth, graciousness, wide cultural interests, and devotion and loyalty to family and friends, perfectly complemented her husband and made it possible for him to achieve great things. It is a tragic lack that Phil’s and my children never knew these two wonderful people who longed so for grandchildren, but we are all immeasurably proud of their accomplishments in the wider world.

## Afterword

In his chapter, Claude Migeon has described with great sensitivity his fondness for my father. I know that Claude filled a special place in Lawson’s life, both as a brilliant colleague and as a surrogate son. I cannot fully express my gratitude to Claude and Barbara for all they did for him, in the happier times, in his lonely, despondent days, and in his illnesses. They supported him when Phil and I were not there and when some of his closest friends could not endure his grief. And how many newlyweds let the boss join them on their honeymoon?

It is hard to believe that Claude and Lawson only knew each other for thirteen years. I have now known Claude and Barbara for fifty years longer. They knew my father better than anyone else alive and have become like family to me. I am so grateful that they have pushed this book to completion. They have my deep appreciation and love.

## Competing interests

The author declares that they have no competing interests.

## Declarations

This article has been published as part of *International Journal of Pediatric Endocrinology* Volume 2014 Supplement 1, 2014: Remembering Doctor Lawson Wilkins: a pioneer of pediatric endocrinology. The full contents of the supplement are available online at http://www.ijpeonline.com/supplements/2014/S1. The next article can be found at http://www.ijpeonline.com/content/2014/S1/S2.
